# Transcriptomic Meta-Analysis and Functional Validation Identify Long Non-Coding RNAs as Modulators of Zika Virus-Mediated Oncolysis in Glioblastoma Multiforme Cell Lines

**DOI:** 10.3390/cells15121088

**Published:** 2026-06-15

**Authors:** Shriya Singh, Martin Gerlein, Allison R. Horvath, Lisa Henderson, Eugene I. Hwang, Roger J. Packer, Chunbo Shao, Youssef A. Kousa, Tamer A. Mansour

**Affiliations:** 1 Division of Neurology, Children’s National Hospital, Washington, DC 20010, USA; ss4996@georgetown.edu (S.S.);; 2 Division of Neurophysiology, Epilepsy, and Critical Care, Children’s National Hospital, Washington, DC 20010, USA; 3 Centers for Precision Medicine and Genomics Research, Children’s National Research and Innovation Campus, 7144 13th Pl NW, Suite 1247, Washington, DC 20012, USA; 4 Section of Infections of the Nervous System, National Institute of Neurological Disorders and Stroke, Bethesda, MD 20892, USA; 5 Division of Oncology, Children’s National Hospital, Washington, DC 20010, USA; 6 Brain Tumor Institute, Children’s National Hospital, Washington, DC 20010, USA; 7 Department of Neurology, The George Washington University School of Medicine and Health Sciences, Washington, DC 20052, USA; 8 Department of Population Health and Reproduction, University of California, Davis, CA 95616, USA; 9 Department of Clinical Pathology, College of Medicine, Mansoura University, Mansoura 35516, Egypt

**Keywords:** glioblastoma, high grade glioma, neuro-oncology, neuroblastoma, zika virus, long non-coding RNAs, transcriptomics

## Abstract

Glioblastoma multiforme (GBM) is the most aggressive primary brain malignancy with limited treatment options and poor clinical outcomes. There is growing interest in using Zika virus as a treatment for GBM due to its selectivity in finding and killing rapidly proliferating neural cells. Several studies reproducibly show that Zika can effectively kill GBM cells. We sought to uncover the molecular mechanisms driving this cytotoxic effect by performing a meta-analysis of transcriptomic studies in which Zika virus was used to kill GBM cells. We integrated four datasets from studies on GBM and added neuroblastoma (NBM) studies as an outgroup comparator. Our analysis identified a shared molecular signature of the Zika-infected GBM cell. Interestingly, GBM cells killed by the Zika virus showed dysregulation of pathways commonly implicated in proliferation and metastasis, including TNF, NF-κB, and p53 signaling. Using a hypothesis-free design, we found several long non-coding RNAs (lncRNAs) that were consistently dysregulated in Zika-infected GBMs, many of which have previously unrecognized roles in cancer cell death. Among this group, we validated four lncRNAs for a role in Zika-mediated oncolysis. We functionally tested *MELTF-AS1*, *TIPARP-AS1*, *NR2F1-AS1*, and *SLC9A3-AS1* in adult GBM cell lines using siRNA-mediated knockdown. Silencing of *MELTF-AS1* augmented Zika-induced cell death, while knockdown of *TIPARP-AS1*, *NR2F1-AS1*, and *SLC9A3-AS1* attenuated oncolysis, identifying lncRNAs whose modulation is associated with altered Zika-mediated cytotoxicity. These findings elucidate candidate mechanisms of Zika oncolysis in GBM cell lines, highlight novel lncRNA targets, and support further exploration of lncRNA modulation as a strategy to enhance oncolytic virotherapy for GBM and related malignancies.

## 1. Introduction

Malignant brain tumors affect approximately 7 per 100,000 individuals annually and account for more than 15,000 deaths in the United States alone. Glioblastoma multiforme (GBM) is the most common and aggressive malignant brain tumor, representing approximately 52% of affected individuals [1]. GBM is classified as an adult-type diffuse glioma, IDH-wildtype, WHO grade 4 under the 2021 WHO Classification of CNS Tumors [2], characterized by rapid cellular proliferation, extensive invasion into surrounding brain tissue, and molecular heterogeneity, all of which contribute to a poor clinical prognosis [3]. The current standard-of-care is to perform maximal surgical resection followed by radiotherapy and temozolomide-based chemotherapy. However, mortality remains high due to treatment resistance and tumor recurrence [4,5,6,7]. As a result, patients diagnosed with GBM face a median survival of 15 months [8]. This challenge is further compounded by evidence suggesting a rising global incidence of GBM [9,10]. Together, these factors highlight the need to explore innovative therapeutic strategies.

Oncolytic viruses are emerging as a promising therapeutic strategy for GBM. In principle, these viruses selectively infect and destroy proliferating tumor cells while largely sparing quiescent healthy tissue [11,12]. Among neurotropic viruses, Zika virus (ZIKV) became the focus of intense scientific investigation after being declared a global public health emergency in 2016, when prenatal infection was linked to severe developmental brain injury and microcephaly. Subsequent research revealed that the same neurotropic properties underlying ZIKV’s teratogenicity—neural cell tropism and preferential replication in proliferative neural cells—might be harnessed therapeutically for targeting and eliminating highly proliferative neural cancers [13,14,15,16,17,18,19]. The phenotypic and transcriptional similarities between the neural stem cells that ZIKV targets in utero and glioblastoma cancer stem cells have focused special attention on the use of ZIKV in GBM. These include high expression of key host factors required for ZIKV binding (including SOX2-integrins) and replication (*MSI1*) in both neural and glioblastoma stem cells [20,21,22]. Furthermore, Zhu et al. (2017) demonstrated that inoculation of a mouse-adapted ZIKV strain into xenografted GBM tumors significantly inhibited tumor growth and improved survival in mice [18].

In contrast to GBM, which originates from glial cells within the central nervous system, neuroblastoma (NBM) is predominantly a pediatric cancer that arises from immature neuroblasts in the developing sympathetic nervous system [23,24,25]. As a result, NBM is distinct in its cellular origin, disease behavior, and molecular profile [24,25]. Despite these differences, both tumor types are of neural lineage, and certain NBM cell lines are highly permissive to ZIKV infection and virally induced cell death, mediated by mechanisms distinct from those in GBM [23,26]. In NBM, susceptibility has been linked to CD24-mediated suppression of basal antiviral signaling, rather than the SOX2-integrin αvβ5 axis that determines ZIKV tropism in GBM stem cells [21,26,27]. Given this mechanistic convergence, we used data from NBM cells as a comparator outgroup to serve as a transcriptomic filter, reasoning that the transcriptional responses shared across both tumor types would reflect the conserved features of ZIKV infection in neural-lineage cells, whereas responses exclusive to GBM would represent tumor-specific oncolytic mechanisms.

lncRNAs are a diverse class of molecular transcripts exceeding 200 nucleotides in length that lack protein-coding potential. Recently, lncRNAs have emerged as regulators of a wide array of biological processes and human diseases [28,29,30]. In the context of human cancer, lncRNAs act as molecular switches that can function as either oncogenic drivers or tumor suppressors depending on the cellular context [31]. In GBM specifically, dysregulated lncRNAs impact the hallmarks of malignancy, including uncontrolled cell proliferation, rapid invasion, angiogenesis, and maintenance of stem cell populations, which drive tumor recurrence and therapy resistance [32,33,34]. By serving as competitive endogenous RNAs (ceRNAs) that sequester and functionally inhibit microRNAs, lncRNAs like *MALAT1*, *HOTAIR*, and *H19* might facilitate tumor progression and confer resistance to standard treatments such as temozolomide (TMZ) and radiotherapy [29,31,35,36]. Consequently, lncRNAs are now recognized as participants in pathogenesis, promising diagnostic biomarkers, and therapeutic targets for precision oncology.

While the protein-coding response to ZIKV is well-documented, the non-coding landscape remains a dark matter of oncolytic biology, potentially containing the primary switches for viral susceptibility. Previous transcriptomic analyses, including those by Zhu et al. and Bonenfant et al., investigated the transcriptional effects of ZIKV infection in GBM and NBM models, respectively [18,23]. These important studies identified molecular dysregulation associated with the antiviral response and cellular stress, but focused on protein-coding genes [18,23]. More recently, Bulstrode et al. (2022) performed transcriptomic profiling of ZIKV-infected malignant neural progenitor cells to assess interferon-mediated effects in GBM [17], and a systematic review by Menezes et al. (2026) identified interferon signaling as the most consistently enriched pathway across ZIKV-infected GBM transcriptomic studies [37]. However, no study has leveraged these datasets to explore the potential oncolytic effect mediated by lncRNA in Zika infection. We integrated these datasets in the current study, which allowed higher statistical power and reduced study-specific effects to enable the identification of conserved molecular signatures associated with ZIKV infection in GBM cells. Further, we functionally tested candidate lncRNAs identified through the meta-analysis and determined whether their modulation influences cancer cell death. These findings provide a framework for understanding transcriptional responses to ZIKV infection in GBM.

## 2. Materials and Methods

### 2.1. Data

Illumina sequencing reads for ZIKV-infected GBM samples and their matched controls were downloaded from SRA for PRJNA399336 (50SE) and PRJNA739733 (50PE) (Table S1). Similarly, infected NBM samples and controls were downloaded for PRJNA630088 (124PE).

### 2.2. Dataset Search and Inclusion Criteria

Studies linked to publicly available data in Gene Expression Omnibus (GEO) Datasets were searched in January 2023 to identify transcriptomic datasets that investigated neural tumor response to ZIKV infection. The search was conducted using the query terms “Zika AND tumor” and filtered for *Homo sapiens* (taxonomy ID: txid9606), yielding 2327 initial records. Applying a filter for expression profiling by high-throughput sequencing reduced this to 15 records for screening (Figure S3). Datasets were evaluated against a standardized set of inclusion criteria: (1) RNA-sequencing capturing total transcriptomic changes (i.e., not targeted panels, microarray, or single-cell platforms); (2) a study design comprising at least two biological replicates each in ZIKV-infected and mock-infected conditions; and (3) infection of a neural tumor or normal brain cell type with a characterized ZIKV strain. Datasets were excluded if raw or processed count data were unavailable, if the experimental design precluded isolation of ZIKV-specific transcriptional effects, or if replication was insufficient for differential expression analysis. Following eligibility assessment, four datasets from three independent studies were retained: two GBM datasets infected with the Dakar and PE243 strains (Zhu et al. and Bulstrode et al. [17,18], respectively) and two NBM datasets infected with the MR766 and PRVABC59 strains (Bonenfant et al. [23]). Given the limited number of ZIKV-GBM transcriptomic studies in the literature, formal sensitivity analysis by leave-one-out was not performed; however, the degree of concordance between the two independently generated GBM datasets (Zhu et al. and Bulstrode et al. [17,18]) provided an internal measure of reproducibility.

### 2.3. Reference Transcriptome

RefSeq gene annotation showed better quantification accuracy compared to Ensembl annotation. However, recent expansion in the number of gene models, specifically those with a smaller size, compromised quantification accuracy [38]. Therefore, the RefSeq release v.110 of gene annotations for *Homo sapiens* were downloaded from NCBI and filtered to exclude transcripts smaller than 250 bp. To adjust for the possible compositional change in genes attributable to viral transcript abundance in infected samples, seven viral genomes were added to the reference as multiple isoforms of one gene. All complete viral genomes were downloaded from the Nucleotide database of NCBI (n [sample size] = 328). The genomes were clustered into groups with a minimum of 95% sequence similarity by cd-hit-est version 4.6 [39]. The longest sequence in each group was selected as a representative.

### 2.4. Differential Expression Analysis

Raw FASTQ sequences were trimmed by Trimmomatic v0.39 to remove adaptors and low-quality sequences [40]. High-quality reads were mapped to the reference transcriptome by bowtie2 v.2.3.4.3 [41]. BAM files were used for quantification of transcript abundance by salmon v.1.9.0 with correction for fragment GC content bias [42]. R v.4.2.1 (2022-06-23) was used for subsequent differential expression analysis [43]. Transcript-level estimates were corrected for the transcript length and summarized for gene-level analysis using tximeta package v.1.14.1 [44]. The DESeq2 package v.1.36.0 was used for differential expression testing using a negative binomial GLM fitting and Wald statistics [45]. The model was adjusted for the biosource in PRJNA739733. Differentially expressed genes were defined by false discovery rate (FDR) less than 0.05. Among the datasets, differentially expressed genes were identified and divided into coding and non-coding groups by custom scripts.

### 2.5. Gene Expression Clustering Analysis

After summarization of the counts per gene, the data were normalized for the library size and its variance was stabilized using the variance stabilizing transformation function in the DESeq2 R package. The counts from the Bulstrode et al. [17] experiment were adjusted for the sample-source batch effect using the limma R package. The counts of the three studied experiments were then combined and adjusted for the study batch effect using the same function. The adjusted count matrix of the differentially expressed genes was used to cluster the differentially expressed genes and assess similarity between samples used in a heatmap and a PCA plot by the pheatmap and plotPCA functions from the pheatmap and DESeq2 R package.

### 2.6. Functional Enrichment Analysis

WebGestalt 2017 [46] was used to test protein-coding differentially expressed genes for pathway enrichment using over-representation testing in KEGG pathways, DrugBank, miRNA targets, and transcription factor targets from the MSigDB database using known protein-coding genes as a background. For each analysis, the minimum and maximum number of genes for a category were set to five and 2000, respectively. The categories were first ranked based on FDR, and then the top 10 most significant categories were selected. This was performed separately for up- and down-regulated categories. The software used Benjamini–Hochberg adjustment to adjust *p* values as it tested multiple gene sets simultaneously. In the analysis of differentially regulated lncRNAs, we reviewed NCBI’s GeneRIF database, and findings pertaining to GBM, NBM, gliomas, and other tumors, were prioritized, as available.

### 2.7. Glioblastoma Cell Cultures

U87 cells (ATCC, Manassas, VA, USA) were cultured in Eagle’s Minimum Essential Medium (EMEM, ATCC) supplemented with 10% Fetal Bovine Serum (FBS, Thermo Fisher Scientific, Waltham, MA, USA), 1% Penicillin/Streptomycin (Sigma-Aldrich, Burlington, MA, USA), and 1% GlutaMAX supplement (Thermo Fisher Scientific). A172 cells (ATCC) were cultured in Dulbecco’s Modified Eagle Medium (DMEM, ATCC) supplemented with 10% Fetal Bovine Serum (FBS, Thermo Fisher Scientific), 1% Penicillin/Streptomycin (Sigma-Aldrich, Burlington, MA, USA), and 1% GlutaMAX supplement (Thermo Fisher Scientific). Cells were maintained in a humidified incubator at 37 °C and 5% CO_2_. These lines were selected as complementary adult IDH1-wildtype GBM models with distinct oncogenic profiles: U87 harbors a homozygous *PTEN* point mutation with constitutive PI3K/AKT activation, wildtype *TP53*, and a methylated *MGMT* promoter with absent *MGMT* expression [47,48], while A172 is characterized by an oncogenic *EGFR* rearrangement producing a constitutively autophosphorylated mutant receptor [49], homozygous *PTEN* deletion [50], wildtype *TP53*, and low *MGMT* expression [51,52]. While both lines share *PTEN* loss, they differ in the mechanism of inactivation and in the presence of oncogenic EGFR signaling in A172, enabling evaluation of lncRNA-mediated ZIKV oncolysis across divergent oncogenic contexts.

### 2.8. ZIKV Titration

ZIKV French Polynesian strain (Nath Lab, NINDS, Bethesda, MD, USA) was expanded in Vero cells (ATCC) by inoculating cells at an MOI of 0.01 in FBS-free DMEM, high glucose, GlutaMAX™ Medium (Thermo Fisher Scientific) and incubating for 72 h at 37 °C and 5% CO_2_. The supernatant containing the virus was mixed with 1× Sucrose-Phosphate-Glutamate, then filtered and stored at −80 °C. Viral titers were quantified by plaque-forming assay. Viral stocks were serially diluted (10^−1^ to 10^−6^), and 100 µL of viral dilution was added to Vero cells in a monolayer in a 24-well plate. After 1 h, 900 µL of overlay media (1% Methylcellulose, 20% PBS, 2% FBS, DMEM with GlutaMAX™, pH 7.5) was added to each well and cells were incubated for 5 days at 37 °C. Cells were fixed in 4% Paraformaldehyde (PFA) and stained with crystal violet dye. Foci were quantified to determine viral titer.

### 2.9. CellTiter-Glo Cell Viability Assay

Following infection/transfection and designated incubation periods, cell viability was quantified using the CellTiter-Glo^®^ Luminescent Cell Viability Assay (Promega, Madison, WI, USA) according to the manufacturer’s instructions. Plates were equilibrated to room temperature for 30 min, followed by the addition of CellTiter-Glo reagent at a 1:4 ratio to the culture medium. After a 20 min incubation, luminescence was measured using a Tecan Spark (Tecan, Zurich, Switzerland) microplate reader with an integration time of 1000 ms per well. Relative luminescence units (RLU) were normalized to untreated control wells.

All experiments were performed with 6–7 replicate wells per condition, and data from a single representative experiment are presented (mean ± SD). Statistical significance was determined by paired, two-tailed Student’s *t*-tests for panels comparing each knockdown condition to the scramble control (Figure 4A,B,D,E), and by one-way matching ANOVA for the ZIKV infection time course (Figure 4C). Data analysis and visualization were performed using GraphPad Prism 11.

### 2.10. ZIKV Infection

U87 and A172 GBM cells were seeded in 24-well plates (1 × 10^5^ cells/well) and 96-well clear-bottom plates (5000 cells/well). Upon reaching approximately 20% confluency, cells were infected with ZIKV at a multiplicity of infection (MOI) of 3. Infections were performed in serum-free and antibiotic-free medium to facilitate viral entry. Following a 3 h incubation at 37 °C, the inoculum was removed and replaced with standard culture medium for the remainder of the experiment.

### 2.11. siRNA-Mediated Gene Silencing

For transient knockdown of candidate lncRNAs, GBM cell lines (U87 and A172) were transfected using Lipofectamine™ RNAiMAX Transfection Reagent (Thermo Fisher Scientific) following the manufacturer’s protocol. In 24-well plates, cells were treated with 25 pmol of siRNA per well, while 96-well plate experiments were scaled proportionally by surface area.

Specific siRNAs targeting *NR2F1-AS1, TIPARP-AS1*, *SLC9A3-AS1*, and *MELTF-AS1* were obtained from Horizon Discovery (Dharmacon, Lafayette, CO, USA), with a non-targeting scramble siRNA utilized as a negative control. siRNA and RNAiMAX were diluted in Opti-MEM™ Reduced Serum Medium (Thermo Fisher Scientific) and complexed at room temperature before being added to the cells. At the indicated time points post-transfection, cytotoxicity was evaluated using the CellTiter-Glo^®^ Luminescent Cell Viability Assay to assess the impact of lncRNA silencing on cell survival.

### 2.12. RNA Extraction and Quantitative Real-Time PCR (qRT-PCR)

Cell pellets were collected at 24, 48, and 72 h post siRNA transfection in GBM cell lines. Total RNA was isolated using TRIzol Reagent (Thermo Fisher Scientific) according to the manufacturer’s protocol. RNA concentration and purity were determined by Nanodrop, and all samples were normalized to a concentration of 20 ng/µL. cDNA synthesis was performed using the SuperScript™ III First-Strand Synthesis SuperMix (Thermo Fisher Scientific).

Quantitative PCR was conducted on a QuantStudio 7 Flex Real-Time PCR System (Thermo Fisher Scientific) using Fast SYBR™ Green Master Mix (Thermo Fisher Scientific) and the specific primers listed in Table S2 (*TIPARP-AS1*, *SLC9A3-AS1*, *NR2F1-AS1*, and *MELTF-AS1*). Target gene expression was normalized to the endogenous control *HPRT*. Each condition was analyzed using three biological replicates. Statistical analysis and data visualization were performed using GraphPad Prism 11.

## 3. Results

### 3.1. ZIKV-Infected GBM and NBM Show Distinct Gene Expression Profiles

To investigate the transcriptional responses underlying ZIKV-mediated oncolysis, we performed a transcriptomic meta-analysis comparing differentially expressed genes (FDR ≤ 0.05) across four datasets from three independent studies. This included two GBM datasets infected by Dakar and PE243 strains (Zhu et al. and Bulstrode et al. [17,18], respectively) and two NBM datasets infected by MR766 and PRVABC59 strains (Bonenfant et al. [23]) (Table S1). By using the NBM datasets as a comparator outgroup, we isolated and subtracted the shared transcriptional response after ZIKV infection in neural-lineage cells, thereby ensuring that the remaining signature reflects highly specialized, GBM-specific oncolytic mechanisms. Furthermore, by combining studies that used different viral strains and cell types, we aimed to identify conserved molecular signatures most likely to reflect fundamental mechanisms of ZIKV oncolysis.

**Figure 1:**
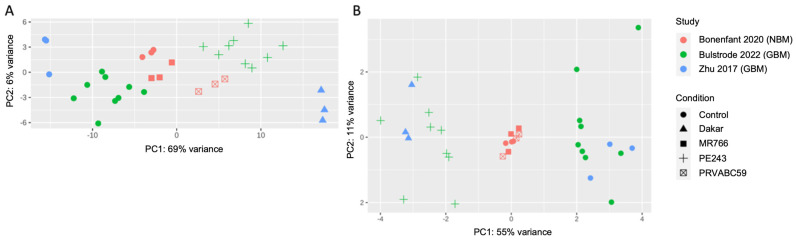
Principal Component Analysis (PCA) of Batch-Corrected Gene Expression Profiles. PCA plots illustrating variance in (**A**) protein-coding and (**B**) non-coding gene expression between control and Zika-infected (Dakar, MR766, PE243, PRVABC59 strains) GBM and NBM cells [17,18,23]. Data were batch-corrected for study-specific effects using limma. Points represent individual samples, with colors indicating the source study and tumor type, while shape defines the infection status and viral strain. Data separate by both tumor types and viral strains.

Principal component analysis (PCA) of batch-corrected gene expression profiles revealed variability between control and ZIKV-infected GBM and NBM samples (Figure 1). The analysis revealed clear segregation between ZIKV-infected and control samples in both GBM and NBM datasets, for both coding (Figure 1A) and non-coding genes (Figure 1B). This pattern was observed across datasets despite differences in cell type and viral strain.

**Figure 2:**
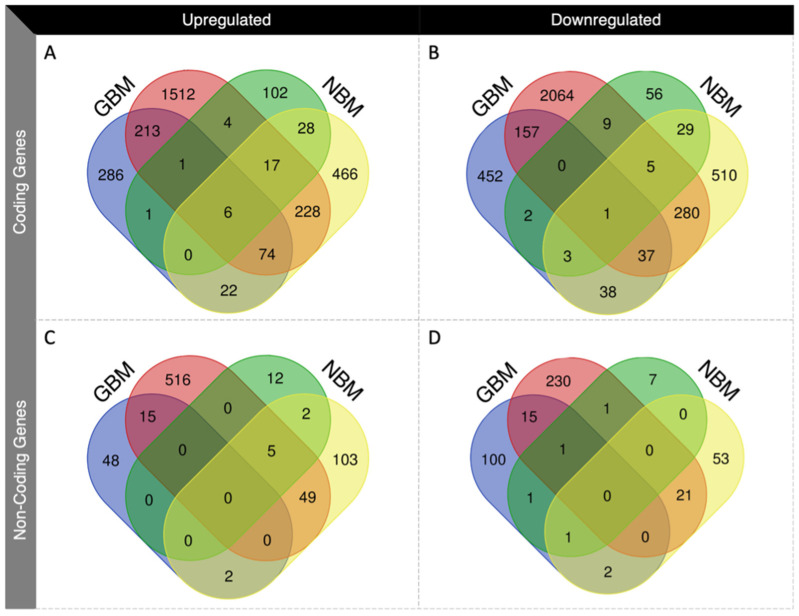
Comparative Analysis of Differentially Expressed Genes in GBM and NBM Studies. GBM datasets are highlighted in blue (Bulstrode et al. 2022 [17]) and red (Zhu et al. 2017 [18]) while NBM datasets are highlighted in green and yellow (Bonenfant et al. 2020 [23]; MR766 and PRVABC59 infection). The intersection of colors indicates genes with shared expression profiles across the different datasets. (**A**,**B**) Venn diagrams showing overlap of differentially expressed protein-coding genes that were (**A**) upregulated or (**B**) downregulated after Zika-infection. (**C**,**D**) Venn diagrams depicting overlap of differentially expressed non-coding genes that were (**C**) upregulated or (**D**) downregulated after Zika-infection. These results show limited overlap across datasets and greater similarity within tumor types.

To further examine similarities and differences in transcriptional responses, we compared differentially expressed genes across datasets. Comparative analysis revealed distinct gene expression profiles between GBM and NBM, with greater overlap within tumor types (Figure 2). Notably, while seven coding genes were shared, no non-coding transcripts reached significance across all four datasets (Table S3A). Given the differences in cell type, developmental origin, and viral strain, this limited overlap was not unexpected and suggests that transcriptional responses to ZIKV infection are largely context dependent. Heatmap analysis supported these transcriptional patterns (Figure S1). Given the limited overlap across datasets, we next focused on identifying consistent transcriptional changes within GBM.

### 3.2. Consensus Protein-Coding Expression Profile in GBM

Meta-analysis of the two GBM studies identified a consensus of 294 upregulated and 195 downregulated protein-coding genes (Figure 2A,B). These gene sets were analyzed using over-representation analysis to assess enrichment across molecular pathways, pharmaceutical agents, miRNA targets, and transcription factor binding sites.

**Figure 3:**
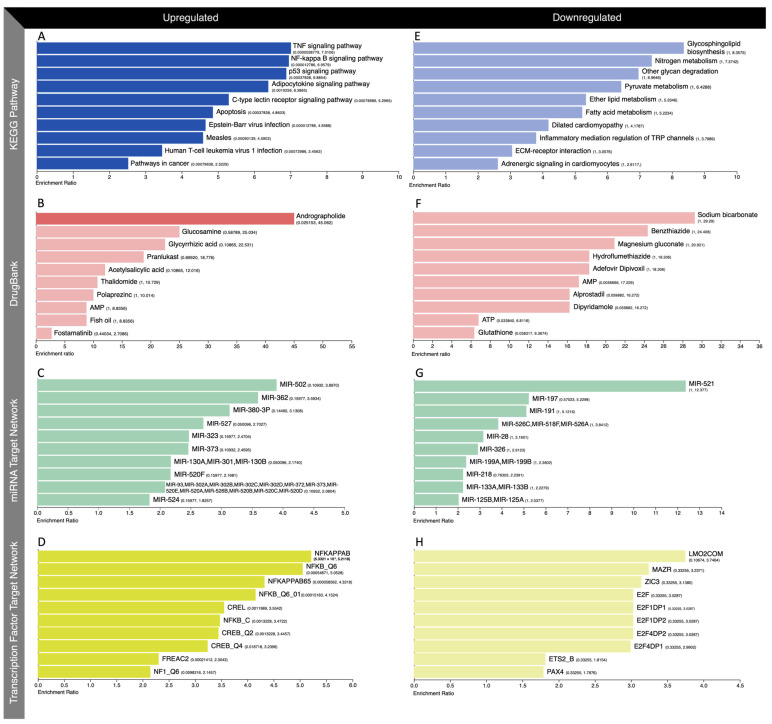
Functional Enrichment Analysis of Dysregulated Protein-Coding Genes in Zika-Infected GBM. Bar graphs show over-representation analysis results of upregulated (**A**–**D**) and downregulated (**E**–**H**) genes in KEGG pathways (**A**,**E**), DrugBank (**B**,**F**), miRNA targets (**C**,**G**), and (**D**,**H**) transcription factor targets. Significance was defined by false discovery rate (FDR) ≤ 0.05. The bars are annotated by *p*-values and enrichment ratios. Together, these findings implicate TNF, NF-κB, and p53 signaling as central mediators of ZIKV-induced oncolysis in GBM, while transcription factor enrichment further supports a pro-inflammatory, anti-proliferative transcriptional shift.

KEGG pathway analysis identified upregulation of genes driving TNF (*p* = 2.9 × 10^−6^), NF-κB (*p* = 1.3 × 10^−5^), and p53 (*p* = 3.8 × 10^−4^) signaling (Figure 3A). Over-representation analysis against pharmaceutical agents in the DrugBank database indicated significant enrichment of upregulated genes for the molecular targets of andrographolide (*p* = 0.025), a natural compound with anti-oncogenic properties (Figure 3B) [53,54,55,56,57,58,59,60]. Enrichment analysis of miRNA targets did not meet FDR threshold, but the top two hits (MIR-502 and MIR-362) have known tumor suppressor functions (Figure 3C) [61,62,63,64,65]. Upregulated genes were significantly enriched in several transcription factor binding sites for NF-κB signaling (*p* = 5.3 × 10^−8^) and CREB (*p* = 1.3 × 10^−3^), both established mediators of cancer-associated inflammation and proliferation, respectively (Figure 3D) [66,67,68]. Over-representation analysis of downregulated protein-coding genes in GBM showed enrichment of multiple metabolic pathways that did not reach statistical significance (Figure 3E–H).

### 3.3. Differential Expression of Long Non-Coding RNAs

**Figure 4:**
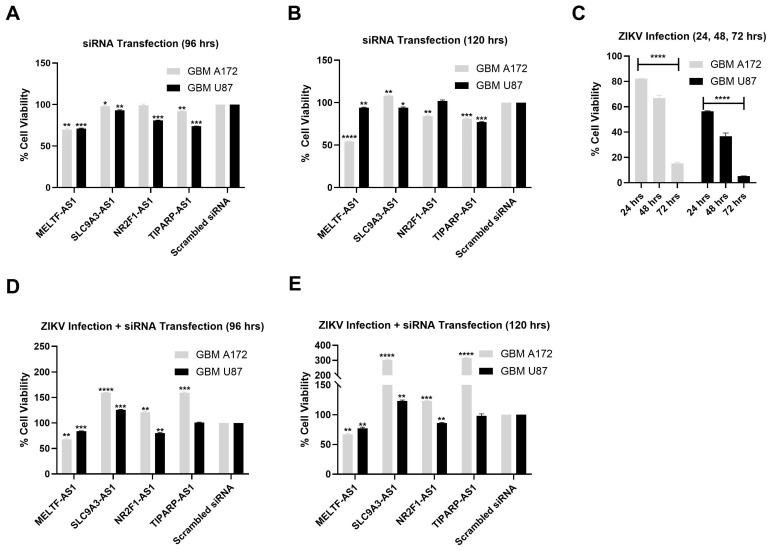
Functional Validation of Candidate lncRNAs in GBM Cell Lines by siRNA-mediated Knockdown. Cell viability was assessed in GBM A172 and U87 cell lines following siRNA-mediated knockdown of *MELTF-AS1*, *SLC9A3-AS1*, *NR2F1-AS1*, and *TIPARP-AS1*, with scrambled siRNA as a negative control. (**A**) Cell viability at 96 h and (**B**) 120 h post-transfection in the absence of ZIKV infection. (**C**) ZIKV alone induces a time-dependent reduction in cell viability in both cell lines at 24, 48, and 72 h post-infection. (**D**) Cell viability following combined ZIKV infection and siRNA knockdown at 96 h and (**E**) 120 h. Viability is expressed as a percentage of the respective scrambled siRNA control (set at 100%). Data represent the mean ± SD of 6–7 replicate wells per condition from a single representative experiment. Statistical significance was assessed by paired two-tailed Student’s *t*-test (**A,B,D,E**) and one-way matching ANOVA (**C**), each comparing treatment conditions to the corresponding control. Significance is denoted as * *p* < 0.05, ** *p* < 0.01, *** *p* < 0.001, **** *p* < 0.0001.

We profiled the total non-coding transcripts to capture additional transcriptional changes associated with ZIKV infection in GBM cells. Among these, 31 lncRNAs (15 upregulated and 16 downregulated) were consistently dysregulated across both GBM studies (Figure 2C,D; Table S3B). These lncRNAs were dysregulated specifically within the GBM cells, with no overlapping dysregulation in the NBM datasets, consistent with tumor-type-specific responses to ZIKV infection. After excluding uncharacterized lncRNAs and pseudogenes, we identified 12 genes for functional literature review. Seven of the 12 genes were found to have documented roles in cancer. Four lncRNAs, *MELTF-AS1*, *TIPARP-AS1, NR2F1-AS1*, and *SLC9A3-AS1*, whose expression patterns were consistent with a potential role in ZIKV-mediated oncolysis, were selected for further validation (Table 1). These candidates were prioritized because they represent the intersection of high statistical conservation in our meta-analysis and diverse functional archetypes (oncogenic vs. tumor-suppressive) in existing cancer literature. These four candidates, spanning both upregulated and downregulated lncRNAs, were functionally validated using small interfering RNA (siRNA)-mediated knockdown, with and without ZIKV infection. (Figure 4; Section 3.4).

### 3.4. Functional Validation of lncRNA Candidates

To investigate the role of identified lncRNA candidates in tumor growth and viral susceptibility, we performed functional validation using siRNA-mediated knockdown in two GBM cell lines (U87 and A172), both in the presence and absence of ZIKV infection. The four candidates spanned both upregulated (*TIPARP-AS1*, *NR2F1-AS1*) and downregulated (*MELTF-AS1*, *SLC9A3-AS1*) lncRNAs based on meta-analysis classification. While siRNA-mediated knockdown is most commonly applied to overexpressed targets, the downregulated candidates were also included to test whether their suppression phenocopies or augments the oncolytic phenotype of ZIKV infection. We first confirmed that ZIKV alone induces a time-dependent oncolytic effect in both cell lines from 24 to 72 h post-infection (Figure 4C). We also evaluated candidate lncRNA expression levels in ZIKV-infected GBM cell lines without siRNA knockdown; no expression change reached statistical significance (Figure S2).

#### 3.4.1. *MELTF-AS1*

*MELTF-AS1* was selected for validation based on its consistent downregulation in ZIKV-infected GBM and its established oncogenic role across multiple tumor types [77]. In that context, *MELTF-AS1* promotes tumor cell survival through additional targets, including the miR-1299/*EGFR* axis and MMP14 [78,79]. We reasoned that if *MELTF-AS1* supports GBM cell survival, its downregulation by ZIKV may contribute to virus-induced cell death, and that further silencing should augment this effect.

Silencing of *MELTF-AS1* consistently reduced cell viability in the GBM A172 and U87 cell lines to 70% and 71%, respectively, at 96 h post-transfection (Figure 4A). In A172 cells, this inhibitory effect persisted through 120 h post-transfection, reducing cell viability to 54% of control, whereas U87 cells showed a partial recovery to 94% at the later timepoint (Figure 4B). When combined with ZIKV infection, *MELTF-AS1* knockdown further decreased viability in both lines after 96 and 120 h, indicating an additive interaction between the lncRNA silencing and ZIKV-mediated oncolysis (Figure 4D).

#### 3.4.2. *TIPARP-AS1*

*TIPARP-AS1* was selected based on its consistent upregulation in ZIKV-infected GBM and its known function as a suppressor of TIPARP/PARP-7. The protein is frequently overexpressed in cancers and aids immune evasion by negatively regulating the type I interferon (IFN) response [80,81]. We hypothesized that ZIKV-induced upregulation of *TIPARP-AS1* alters TIPARP/PARP-7 function, potentially modulating IFN signaling in GBM cells and contributing to viral cytotoxicity. Under this model, silencing *TIPARP-AS1* would disrupt this interaction, leading to changes in IFN responses and ZIKV-mediated oncolysis.

*TIPARP-AS1* silencing resulted in a slight decrease in viability in U87 cells at 96 h post-transfection (74% of control), while A172 cells showed a smaller but significant reduction (92% of control) (Figure 4A). Under ZIKV-infected conditions, *TIPARP-AS1* silencing led to a cell line-specific response: U87 showed no apparent change, while A172 cells exhibited increased cell viability at 96 h (159% of control) (Figure 4D). Notably, at 120 h, *TIPARP-AS1* silencing in A172 cells increased viability to 314% of control (Figure 4E). No change was observed in U87 cells under the same conditions. These results are consistent with *TIPARP-AS1* functioning as a pro-oncolytic facilitator whose loss attenuates ZIKV killing in a cell-line-dependent manner.

#### 3.4.3. *NR2F1-AS1*

*NR2F1-AS1* was selected based on its consistent upregulation in ZIKV-infected GBM and its role as a tumor suppressor in NBM based on published literature, where it targets the miR-493/TRIM2 axis to suppress cell proliferation and migration and increase apoptosis [69]. Given this pro-apoptotic profile, we reasoned that its upregulation in ZIKV-infected GBM might facilitate tumor cell death, and that silencing it would reduce oncolytic efficacy.

Knockdown of *NR2F1-AS1* resulted in a modest decrease in viability in U87 cells at 96 h post-transfection (81% of control), while A172 cells remained largely unaffected (Figure 4A). Under ZIKV-infected conditions, *NR2F1-AS1* silencing increased viability in A172 cells to 121% of control at 96 h (Figure 4D), with a similar increase persisting at 120 h (Figure 4E), while U87 cells showed a modest decline in viability (80% at 96 h post-infection). These findings suggest that *NR2F1-AS1* may facilitate ZIKV-mediated killing in a cell-line-dependent manner, and that its loss attenuates oncolytic efficacy.

#### 3.4.4. *SLC9A3-AS1*

*SLC9A3-AS1* was selected based on its consistent downregulation in ZIKV-infected GBM and its characterized oncogenic function in nasopharyngeal carcinoma, where loss of *SLC9A3-AS1* reduces proliferation and induces apoptosis through the miR-486-5p/E2F6 axis [72]. We reasoned that downregulation of *SLC9A3-AS1* by ZIKV may represent a pro-oncolytic event, and that silencing should similarly reduce GBM cell viability. While *SLC9A3-AS1* was classified as downregulated across both GBM datasets in the meta-analysis, expression profiling by qPCR did not reveal statistically significant changes in either cell line following ZIKV infection (Figure S2).

Silencing *SLC9A3-AS1* alone produced small but statistically significant changes in cell viability in both GBM cell lines (Figure 4A,B). When combined with ZIKV infection, however, *SLC9A3-AS1* knockdown surprisingly increased cell viability to 159% and 126% in A172 and U87 cells, respectively, at 96 h (Figure 4D). This effect was more pronounced at 120 h, with A172 viability reaching 303% of control and U87 viability increasing to 123%. This suggests that *SLC9A3-AS1*, despite its oncogenic classification in the meta-analysis, functions as a pro-oncolytic element in the context of ZIKV infection.

## 4. Discussion

Our transcriptomic meta-analysis of ZIKV-infected GBM, using NBM as a comparative outgroup, identified a conserved set of dysregulated molecular networks and several candidate lncRNAs with potential roles in ZIKV-mediated oncolysis. Functional validation indicated that lncRNA modulation can alter viral killing efficiency in GBM cell lines, with distinct effects depending on the gene and cellular context. These findings advance our mechanistic understanding of ZIKV oncolysis and identify specific lncRNAs as candidate targets for further investigation.

**Figure 5:**
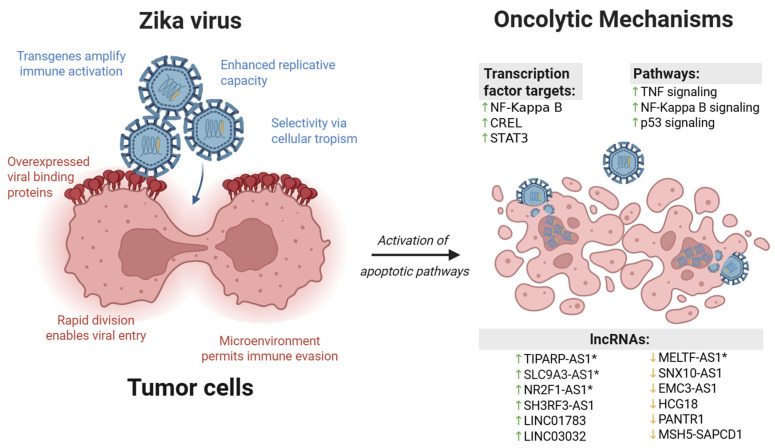
Proposed Model of Zika’s Oncolytic Mechanisms. Schematic illustrating how tumor susceptibilities and viral factors converge to mediate Zika’s oncolytic effects in GBM. Viral-specific factors (blue) include: (1) viral transgenes capable of modulating immune activation; (2) fine-tuning of infectious dose and/or replication rate within the tumor cell; and (3) cellular specificity via innate tropism. Tumor-specific factors (red) include: (1) overexpression of viral binding proteins; (2) rapid cell division; and (3) immune evasion within the tumor microenvironment. Our meta-analysis suggests a virally induced oncolytic impact mediated through differential regulation of the selected (1) transcription factors; (2) gene signaling pathways; and (3) lncRNAs (asterisks denote lncRNAs with experimentally assigned functional direction: *TIPARP-AS1*, *SLC9A3-AS1*, *NR2F1-AS1*, *MELTF-AS1*). Arrows indicate up/downregulation following infection with ZIKV. Created with BioRender.com.

We identified upregulation of genes driving canonical tumor pathways, including TNF, NF-κB, and p53 signaling pathways and a refined list of twelve lncRNAs with altered expression in ZIKV-infected GBM (Table 1; Figure 5). These findings extend the work of Zhu et al. and Bulstrode et al., who highlighted the involvement of inflammatory and immune pathways in ZIKV oncolysis in GBM [17,18]. Both studies found significant enrichment of interferon-stimulated genes and demonstrated that ZIKV’s antitumor efficacy can be enhanced through inhibition of type I interferons or JAK/STAT signaling.

The comparative analysis between GBM and NBM (Figure 2) revealed substantially more transcriptional differences than similarities, consistent with distinct cellular origins and molecular profiles of these tumor types. Despite these molecular differences, it is interesting that the cell-killing capacity for ZIKV in these neural cancers is high. This tumor-type specificity suggests that ZIKV-induced transcriptional responses may depend on the underlying cellular context rather than representing a uniform response across neural-derived tumors.

### 4.1. MELTF-AS1, TIPARP-AS1, NR2F1-AS1, and SLC9A3-AS1 as Modulators of ZIKV Oncolysis

Despite being known regulators of cancer progression in GBM, lncRNAs remain a critical yet understudied component of the ZIKV-GBM transcriptome, and their involvement in ZIKV-mediated oncolysis has not been systematically characterized. The functional data presented here suggest that several lncRNAs dysregulated during ZIKV infection play active roles in modulating viral killing efficiency, with distinct effects depending on the gene and cellular context.

siRNA-mediated silencing of *MELTF-AS1* reduced cell viability both alone and in combination with ZIKV infection, suggesting a pro-survival role in GBM, where its natural downregulation during infection may contribute to viral killing. The additional decrease in viability observed when silencing is combined with ZIKV infection suggests additive or partially overlapping mechanisms of cell death, although distinguishing between these will require further study. Therapeutic targeting of *MELTF-AS1* through siRNA, antisense oligonucleotides, or small molecule approaches could therefore serve as a complementary strategy to augment ZIKV-mediated oncolysis.

For *TIPARP-AS1*, the functional data are consistent with the mechanistic rationale outlined above. Its upregulation by ZIKV, combined with the observation that silencing it reduces viral killing efficiency, suggests that *TIPARP-AS1* induction may be functionally indispensable to the oncolytic program. This may occur through suppression of *TIPARP*/PARP-7, given that antisense lncRNAs commonly repress their sense counterparts, though direct suppression of PARP-7 by *TIPARP-AS1* has not yet been demonstrated in the published literature [70]. Under this model, PARP-7 loss would disinhibit type I IFN signaling at two levels: restoring IFN production through loss of TBK1 MARylation, which permits IRF3-mediated IFN transcription, and enhanced downstream IFN signaling through stabilization of STAT1/STAT2 against autophagic degradation [81,82]. In the context of ZIKV-mediated oncolysis, restored type I IFN signaling may therefore contribute to tumor cell death through direct antiproliferative and proapoptotic effects on GBM cells, as well as broader immunostimulatory signaling within the tumor microenvironment [17,82,83]. The pronounced increase in cell viability seen with *TIPARP-AS1* silencing in A172 cells at 120 h suggests this pathway may be particularly active in that cell line, pointing to cell-line-specific differences in IFN-mediated oncolysis that warrant further investigation.

*NR2F1-AS1* silencing produced a smaller but directionally similar effect to *TIPARP-AS1*, partially attenuating ZIKV oncolysis in A172 cells, though the opposing response observed in U87 cells indicates a cell-line-dependent effect. *NR2F1-AS1* is a known mediator of tumor cell dormancy through regulation of the miR-485-5p/NR2F1 and miR-493/TRIM2 axes [69,84,85], and its upregulation by ZIKV may help shift surviving GBM cells toward a quiescent state, reducing their capacity to repopulate after initial viral cytotoxicity. The more modest effect size compared to *TIPARP-AS1* suggests *NR2F1-AS1* may play a supporting rather than primary role in the killing program, though further studies would be needed to determine whether these lncRNAs act through independent or overlapping pathways.

The behavior of *SLC9A3-AS1* highlights a critical phenomenon of transcriptional reprogramming during viral infection. Although characterized as an oncogene in multiple cancer types, notably nasopharyngeal carcinoma, lung cancer, and colon cancer [72,86,87], its silencing increased viability in ZIKV-infected GBM cells, contradicting the initial prediction. This functional inversion reflects the tissue-specific role of its downstream target rather than active viral reprogramming of *SLC9A3-AS1* itself. In nasopharyngeal carcinoma, *SLC9A3-AS1* sponges miR-486-5p to produce oncogenic effects [72]; however, in GBM, miR-486-5p is itself oncogenic, sustaining stem cell survival by targeting *PTEN* and *FoxO1* [88], such that its sponging by *SLC9A3-AS1* would instead produce pro-apoptotic outcomes. This context-dependency is consistent with broader observations that lncRNA function is highly tissue- and context-specific and underscores a practical limitation of using cross-cancer literature to predict function in a new setting. From a therapeutic standpoint, these data suggest that forced overexpression, rather than silencing, of *SLC9A3-AS1* may be the more appropriate strategy to augment ZIKV efficacy, though this would require formal efficacy validation in primary GBM cells and in vivo models, alongside careful consideration of safety given *SLC9A3-AS1*’s established oncogenic activity in other tumor contexts.

Collectively, these findings indicate that lncRNA expression in ZIKV-infected GBM may modulate viral killing efficiency. The differing functional effects observed across the four candidates highlight the complexity of lncRNA involvement in this process; *MELTF-AS1* silencing enhanced oncolysis, whereas *TIPARP-AS1* and *NR2F1-AS1* silencing reduced it, and *SLC9A3-AS1* showed a context-dependent response. While further studies in primary GBM cells and in vivo models will be needed to determine clinical relevance, these results provide a foundational framework for investigating lncRNA modulation as a complementary strategy to improve ZIKV-based oncolytic approaches.

Beyond effects on tumor cell viability, the lncRNAs identified here may also influence ZIKV replication dynamics within GBM cells. ZIKV replication in GBM stem cells is facilitated by the RNA-binding protein MSI1, which binds the viral genome and promotes intracellular replication, while SOX2 governs viral tropism by upregulating integrin αvβ5 expression and suppressing baseline interferon-stimulated gene expression [21,22]. Type I IFN signaling is known to directly restrict ZIKV replication in this context [17]. Given that *TIPARP-AS1* upregulation may modulate IFN responses, and that the broader lncRNA dysregulation identified in our meta-analysis likely influences the cellular environment permissive to viral replication, whether these lncRNAs contribute to the balance between viral replication efficiency and tumor cell death represents an important question for future investigation.

### 4.2. Additional Findings

A noteworthy secondary finding is the enrichment of andrographolide targets among ZIKV-upregulated genes. Andrographolide induces cell cycle arrest, apoptosis, and suppresses migration in GBM [53,54,55]. The activation of p53 signaling, which is a hallmark of our meta-analysis, operates in a reciprocal circuit with the downregulation of pro-survival lncRNAs like *MELTF-AS1*, creating a multi-layered apoptotic response specific to ZIKV-infected GBM cells. p53 has been shown to transcriptionally repress oncogenic lncRNAs through direct promoter binding, forming feedback loops that amplify tumor-suppressive signaling [89]. Such p53-lncRNA circuits have been increasingly recognized as amplifiers of the cellular stress response in cancer [90]. Within this framework, ZIKV-induced p53 activation may contribute to the observed downregulation of *MELTF-AS1*; the resulting loss of *MELTF-AS1*-mediated signaling, including the collapse of the downstream EGFR and MMP14 survival networks, would in turn reinforce p53-dependent apoptosis [78,79,90].

Interestingly, andrographolide and ZIKV infection share downstream regulation of CREB, TNF, *NFKB1*, and *NFKB2*, although the direction of gene expression changes differs [55,91]. Mechanistically, andrographolide inhibits NF-κB signaling through covalent modification of Cys62 on the p50 subunit (encoded by *NFKB1*), directly blocking DNA binding activity [92,93]. Separately, the p100/p52 subunit (encoded by *NFKB2*) mediates the non-canonical NF-κB pathway, which has independently been implicated in GBM invasion and proliferation [94,95]. The overlap between andrographolide’s known targets and the NF-κB-associated transcriptional changes identified in our meta-analysis suggests convergence on shared regulatory nodes through mechanistically distinct routes, and the therapeutic utility of this relationship warrants further investigation.

### 4.3. A Convergent Model of lncRNA-Mediated Oncolysis in GBM

The efficacy of ZIKV-mediated oncolysis appears to rely on a coordinated collapse of the GBM pro-survival network, orchestrated by both protein-coding genes and lncRNAs. Our meta-analysis identifies a “molecular pincer” effect: while ZIKV activates canonical cell death pathways such as TNF and p53 signaling, it simultaneously dysregulates specific lncRNAs that function as essential pro-oncolytic facilitators or pro-survival inhibitors. For instance, the consistent downregulation of *MELTF-AS1*, an oncogene known to drive tumor progression through the miR-485-5p/*MMP14* axis in GBM and miR-1299/*EGFR* axes in other cancer types [74,78,79], directly complements the upregulation of p53 targets, effectively removing the “brakes” on apoptosis. Furthermore, the induction of *TIPARP-AS1* suggests a regulatory feedback loop where lncRNA-mediated suppression of TIPARP/PARP-7 relieves inhibition of the type I IFN response, thereby amplifying the very inflammatory pathways (NF-κB and TNF) identified in our consensus signature. This convergence suggests that lncRNAs do not merely respond to infection but actively tune the tumor’s molecular landscape to permit efficient viral killing as summarized in our integrated oncolytic model (Figure 5).

### 4.4. Strengths/Limitations

The strengths of this study include the integration of multiple transcriptomic datasets spanning different viral strains and tumor types to identify shared and context-specific transcriptional responses to ZIKV infection. In addition, the combination of meta-analysis with functional validation in GBM cell lines provides an initial framework for linking transcriptional changes to cellular outcomes. However, limitations should be considered. The meta-analysis was restricted to four publicly available datasets, reflecting the limited availability of ZIKV-infected neural tumor transcriptomic data, which reduces statistical power and increases the influence of individual studies. Further, the inclusion of different viral strains and cell models introduces biological and technical heterogeneity that may have limited the ability to fully separate study-specific effects from shared biological signals. The inclusion of NBM datasets as a comparator outgroup is intended to delineate GBM-specific transcriptional responses from general features of ZIKV infection in neural-lineage cells. We acknowledge that comparing distinct tumor entities, utilizing multiple viral strains, and merging diverse data sources introduces technical and biological heterogeneity. Therefore, NBM data must not be interpreted as informing or predicting glioblastoma biology directly, but rather as an outgroup control to narrow down candidate networks specific to the GBM context. Only two established cell lines were investigated, providing a limited view of the molecular heterogeneity of primary GBM tumors. Finally, while several lncRNAs were associated with altered cell viability in the context of ZIKV infection, these findings do not establish causality or define the underlying molecular mechanisms. Further studies will be required to validate and extend these findings.

### 4.5. Clinical Translatability

Despite its promising oncolytic potential, the clinical translation of ZIKV-based therapies raises important safety considerations that must be addressed prior to human application. Naturally circulating, non-attenuated ZIKV poses well-documented risks of congenital Zika syndrome in fetuses exposed during pregnancy, including fetal microcephaly and developmental malformations, as well as Guillain-Barré syndrome in infected adults [14,96]. These risks necessitate that any therapeutic application be delivered under controlled conditions to a carefully screened patient population. To address these concerns, several groups have engineered attenuated ZIKV strains with reduced neurovirulence while preserving oncolytic activity. Notably, Zhou and colleagues utilized a miRNA-modified recombinant ZIKV in an orthotopic model of glioma in mice, demonstrating tumor-selective replication with markedly reduced infectivity in normal neural progenitor cells, representing a promising strategy to decouple therapeutic efficacy from neurovirulence risk [97]. Together, these strategies highlight a viable path toward safer clinical translation, though formal safety profiling in immune-competent models and rigorous patient selection criteria will remain essential prerequisites before human application can be considered.

A related safety consideration is whether non-malignant astrocytes may constitute collateral targets of ZIKV oncolysis. Primary human astrocytes are permissive to ZIKV infection, with AXL serving as a key entry receptor [98,99,100], indicating that non-malignant astrocytes could, in principle, be infected. However, ZIKV’s preferential oncolytic tropism for GBM stem cells, governed primarily by the SOX2–integrin αvβ5 axis, has been demonstrated relative to differentiated neurons and normal neural cells [18,21], providing a basis for relative tumor selectivity. The susceptibility of astrocytes within the tumor stroma may be more complex, as tumor-associated reactive astrocytes express high levels of AXL [101], undergo proliferative reprogramming, and may retain partial stem-like features [102]. Whether primary or reactive astrocytes represent collateral targets of ZIKV oncolysis warrants direct investigation as part of any preclinical safety assessment.

## 5. Conclusions

This study demonstrates that lncRNA expression is a functionally relevant component of ZIKV-mediated oncolysis in GBM and suggests gene-specific strategies for lncRNA-based oncolytic ZIKV treatment. Our transcriptomic meta-analysis identified consistently dysregulated pathways, including TNF, NF-κB, and p53 signaling, alongside several candidate lncRNAs that modulate ZIKV’s oncolytic efficiency. Functional validation indicated that *MELTF-AS1* may act as a pro-survival oncogenic factor whose silencing augments ZIKV-induced cell death, while *TIPARP-AS1*, *NR2F1-AS1*, and *SLC9A3-AS1* were identified as candidate pro-oncolytic facilitators whose knockdown was associated with attenuated viral killing in cell line models, providing a comprehensive schematic for ZIKV-mediated oncolysis (Figure 5). Collectively, these findings support further investigation of targeted lncRNA modulation as a strategy to enhance ZIKV oncolytic efficiency and address treatment resistance in GBM.

## Figures and Tables

**Table 1:** Literature Review for Possible/Known Functions of GBM-dysregulated lncRNA. Expression indicates the type of dysregulation detected in our meta-analysis.

Gene	Expression in GBM	Literature Review		
		Tumor Type	Possible/Known Function	Ref.
*NR2F1-AS1*	Upregulated	Neuroblastoma	Tumor Suppressor: targets miR-493/TRIM2 axis. Upregulation suppresses cell proliferation and migration, while increasing apoptosis.	[69]
*LINC03032*	Upregulated	-	-	-
*TIPARP-AS1*	Upregulated	Breast Cancer	Regulates TIPARP-mediated AHR Signaling.	[70]
*SH3RF3-AS1*	Upregulated	-	-	-
*LINC01783*	Upregulated	Tongue Squamous Cell Carcinoma	Oncogene: targets miR-199b-5p. Upregulation promoted cell proliferation and metastasis.	[71]
*SNX10-AS1*	Downregulated	-	-	-
*SLC9A3-AS1*	Downregulated	Nasopharyngeal Carcinoma	Oncogene: targets miR-486-5p/E2F6 axis. Loss of *SLC9A3-AS1* reduced cell proliferation and metastasis, while inducing apoptosis in vitro; reduced tumor growth in vivo.	[72]
*EMC3-AS1*	Downregulated	-	-	-
*HCG18*	Downregulated	Anaplastic Glioma	Tumor Suppressor/Protective Factor. Downregulation associated with increased tumor grade.	[73]
*MELTF-AS1*	Downregulated	Glioblastoma Multiforme	Oncogene: targets miR-485-5p/MMP14 axis. Downregulation suppresses tumor growth and metastasis, while inducing apoptosis; silencing represses tumor growth in vivo.	[74]
*PANTR1*	Downregulated	Glioblastoma Multiforme	Oncogene: targets POU3F3.	
			Loss of *PANTR1* decreased proliferation, colony formation, and viability.	[75]
			Silencing decreased expression of pro-angiogenesis factors (bFGF, VEGFA, bFGFR, and Angio).	[76]
*MSH5-SAPCD1*	Downregulated	-	-	-

## Data Availability

The original contributions presented in the study are included in the article/Supplementary Material; further inquiries can be directed to the corresponding authors.

## Supplementary Materials

The following supporting information can be downloaded at: https://www.mdpi.com/article/10.3390/cells15121088/s1, Figure S1: Heatmaps of coding and non-coding genes; Figure S2: Differential Expression of Candidate lncRNAs in GBM A172 and U87 Cell Lines Following ZIKV Infection; Figure S3: PRISMA flowchart for GEO DataSets search and transcriptomic dataset selection; Table S1: Overview of meta-analysis studies; Table S2: lncRNA primers used for quantitative real-time PCR; Table S3: Differentially Expressed Genes.

## References

[1] Price M., Ballard C., Benedetti J.R., Kruchko C., Barnholtz-Sloan J.S., Ostrom Q.T. (2025). CBTRUS Statistical Report: Primary Brain and Other Central Nervous System Tumors Diagnosed in the United States in 2018–2022. Neuro-Oncol..

[2] Louis D.N., Perry A., Wesseling P., Brat D.J., Cree I.A., Figarella-Branger D., Hawkins C., Ng H.K., Pfister S.M., Reifenberger G. (2021). The 2021 WHO Classification of Tumors of the Central Nervous System: A Summary. Neuro-Oncol..

[3] Omuro A., DeAngelis L.M. (2013). Glioblastoma and Other Malignant Gliomas: A Clinical Review. JAMA.

[4] Sung H., Ferlay J., Siegel R.L., Laversanne M., Soerjomataram I., Jemal A., Bray F. (2021). Global Cancer Statistics 2020: GLOBOCAN Estimates of Incidence and Mortality Worldwide for 36 Cancers in 185 Countries. CA Cancer J. Clin..

[5] Habashy K.J., Mansour R., Moussalem C., Sawaya R., Massaad M.J. (2022). Challenges in Glioblastoma Immunotherapy: Mechanisms of Resistance and Therapeutic Approaches to Overcome Them. Br. J. Cancer.

[6] Dymova M.A., Kuligina E.V., Richter V.A. (2021). Molecular Mechanisms of Drug Resistance in Glioblastoma. Int. J. Mol. Sci..

[7] Nair S., Mazzoccoli L., Jash A., Govero J., Bais S.S., Hu T., Fontes-Garfias C.R., Shan C., Okada H., Shresta S. (2021). Zika Virus Oncolytic Activity Requires CD8+ T Cells and Is Boosted by Immune Checkpoint Blockade. JCI Insight.

[8] Grochans S., Cybulska A.M., Siminska D., Korbecki J., Kojder K., Chlubek D., Baranowska-Bosiacka I. (2022). Epidemiology of Glioblastoma Multiforme-Literature Review. Cancers.

[9] Lin D., Wang M., Chen Y., Gong J., Chen L., Shi X., Lan F., Chen Z., Xiong T., Sun H. (2021). Trends in Intracranial Glioma Incidence and Mortality in the United States, 1975–2018. Front. Oncol..

[10] Grech N., Dalli T., Mizzi S., Meilak L., Calleja N., Zrinzo A. (2020). Rising Incidence of Glioblastoma Multiforme in a Well-Defined Population. Cureus.

[11] Liu J., Wang Y., Su S., Cheng G., Zhao H., Sun J., Sun G., Li F., Hui R., Liu M. (2025). Oncolytic Viruses in Glioblastoma: Clinical Progress, Mechanistic Insights, and Future Therapeutic Directions. Cancers.

[12] Lawler S.E., Speranza M.C., Cho C.F., Chiocca E.A. (2017). Oncolytic Viruses in Cancer Treatment: A Review. JAMA Oncol..

[13] WHO (2016). WHO Statement on the First Meeting of the International Health Regulations (2005).

[14] Freitas D.A., Souza-Santos R., Carvalho L.M.A., Barros W.B., Neves L.M., Brasil P., Wakimoto M.D. (2020). Congenital Zika Syndrome: A Systematic Review. PLoS ONE.

[15] Li C., Xu D., Ye Q., Hong S., Jiang Y., Liu X., Zhang N., Shi L., Qin C.F., Xu Z. (2016). Zika Virus Disrupts Neural Progenitor Development and Leads to Microcephaly in Mice. Cell Stem Cell.

[16] Tang H., Hammack C., Ogden S.C., Wen Z., Qian X., Li Y., Yao B., Shin J., Zhang F., Lee E.M. (2016). Zika Virus Infects Human Cortical Neural Progenitors and Attenuates Their Growth. Cell Stem Cell.

[17] Bulstrode H., Girdler G.C., Gracia T., Aivazidis A., Moutsopoulos I., Young A.M.H., Hancock J., He X., Ridley K., Xu Z. (2022). Myeloid Cell Interferon Secretion Restricts Zika Flavivirus Infection of Developing and Malignant Human Neural Progenitor Cells. Neuron.

[18] Zhu Z., Gorman M.J., McKenzie L.D., Chai J.N., Hubert C.G., Prager B.C., Fernandez E., Richner J.M., Zhang R., Shan C. (2017). Correction: Zika Virus Has Oncolytic Activity against Glioblastoma Stem Cells. J. Exp. Med..

[19] Zhou C., Chen Q., Chen Y., Qin C.F. (2023). Oncolytic Zika Virus: New Option for Glioblastoma Treatment. DNA Cell Biol..

[20] Lee J.H., Lee J.H. (2018). The Origin-of-Cell Harboring Cancer-Driving Mutations in Human Glioblastoma. BMB Rep..

[21] Zhu Z., Mesci P., Bernatchez J.A., Gimple R.C., Wang X., Schafer S.T., Wettersten H.I., Beck S., Clark A.E., Wu Q. (2020). Zika Virus Targets Glioblastoma Stem Cells through a SOX2-Integrin Alpha(v)Beta(5) Axis. Cell Stem Cell.

[22] Chavali P.L., Stojic L., Meredith L.W., Joseph N., Nahorski M.S., Sanford T.J., Sweeney T.R., Krishna B.A., Hosmillo M., Firth A.E. (2017). Neurodevelopmental Protein Musashi-1 Interacts with the Zika Genome and Promotes Viral Replication. Science.

[23] Bonenfant G., Meng R., Shotwell C., Badu P., Payne A.F., Ciota A.T., Sammons M.A., Berglund J.A., Pager C.T. (2020). Asian Zika Virus Isolate Significantly Changes the Transcriptional Profile and Alternative RNA Splicing Events in a Neuroblastoma Cell Line. Viruses.

[24] Tolbert V.P., Matthay K.K. (2018). Neuroblastoma: Clinical and Biological Approach to Risk Stratification and Treatment. Cell Tissue Res..

[25] Ponzoni M., Bachetti T., Corrias M.V., Brignole C., Pastorino F., Calarco E., Bensa V., Giusto E., Ceccherini I., Perri P. (2022). Recent Advances in the Developmental Origin of Neuroblastoma: An Overview. J. Exp. Clin. Cancer Res..

[26] Mazar J., Li Y., Rosado A., Phelan P., Kedarinath K., Parks G.D., Alexander K.A., Westmoreland T.J. (2018). Zika Virus as an Oncolytic Treatment of Human Neuroblastoma Cells Requires CD24. PLoS ONE.

[27] Kedarinath K., Fox C.R., Crowgey E., Mazar J., Phelan P., Westmoreland T.J., Alexander K.A., Parks G.D. (2022). CD24 Expression Dampens the Basal Antiviral State in Human Neuroblastoma Cells and Enhances Permissivity to Zika Virus Infection. Viruses.

[28] Maruyama R., Suzuki H. (2012). Long Noncoding RNA Involvement in Cancer. BMB Rep..

[29] Fatima R., Akhade V.S., Pal D., Rao S.M. (2015). Long Noncoding RNAs in Development and Cancer: Potential Biomarkers and Therapeutic Targets. Mol. Cell. Ther..

[30] Ye J., Li H., Wei J., Luo Y., Liu H., Zhang J., Luo X. (2020). Risk Scoring System Based on lncRNA Expression for Predicting Survival in Hepatocellular Carcinoma with Cirrhosis. Asian Pac. J. Cancer Prev..

[31] Pei D., Zhang D., Guo Y., Chang H., Cui H. (2025). Long Non-Coding RNAs in Malignant Human Brain Tumors: Driving Forces Behind Progression and Therapy. Int. J. Mol. Sci..

[32] Hashemi M., Mousavian Roshanzamir S., Orouei S., Daneii P., Raesi R., Zokaee H., Bikarannejad P., Salmani K., Khorrami R., Deldar Abad Paskeh M. (2024). Shedding Lighton Function of Long Non-Coding RNAs (lncRNAs) in Glioblastoma. Noncoding RNA Res..

[33] Tremante E., Diaz Mendez A.B., Rizzo M.G. (2025). The Role of LncRNAs in Radio- and Chemoresistance of Glioblastoma: Prognostic or Therapeutic?. Curr. Oncol..

[34] Shahzad U., Krumholtz S., Rutka J.T., Das S. (2021). Noncoding RNAs in Glioblastoma: Emerging Biological Concepts and Potential Therapeutic Implications. Cancers.

[35] Chae Y., Roh J., Kim W. (2021). The Roles Played by Long Non-Coding RNAs in Glioma Resistance. Int. J. Mol. Sci..

[36] Guo Y., Xie Y., Luo Y. (2022). The Role of Long Non-Coding RNAs in the Tumor Immune Microenvironment. Front. Immunol..

[37] Menezes D., Reis C.R., Mamede I., Geddes V.E.V., de Souza R.P., Aguiar R.S. (2026). Transcriptomic Profile of Glioblastoma Cells Infected with Zika Virus: A Systematic Review and Pathway Analysis. Viruses.

[38] Chisanga D., Liao Y., Shi W. (2022). Impact of Gene Annotation Choice on the Quantification of RNA-seq Data. BMC Bioinform..

[39] Fu L., Niu B., Zhu Z., Wu S., Li W. (2012). CD-HIT: Accelerated for Clustering the next-Generation Sequencing Data. Bioinformatics.

[40] Bolger A.M., Lohse M., Usadel B. (2014). Trimmomatic: A Flexible Trimmer for Illumina Sequence Data. Bioinformatics.

[41] Langmead B., Salzberg S.L. (2012). Fast Gapped-Read Alignment with Bowtie 2. Nat. Methods.

[42] Patro R., Duggal G., Love M.I., Irizarry R.A., Kingsford C. (2017). Salmon Provides Fast and Bias-Aware Quantification of Transcript Expression. Nat. Methods.

[43] R Core Team (2022). R: A Language and Environment for Statistical Computing.

[44] Love M.I., Soneson C., Hickey P.F., Johnson L.K., Pierce N.T., Shepherd L., Morgan M., Patro R. (2020). Tximeta: Reference Sequence Checksums for Provenance Identification in RNA-Seq. PLoS Comput. Biol..

[45] Love M.I., Huber W., Anders S. (2014). Moderated Estimation of Fold Change and Dispersion for RNA-Seq Data with DESeq2. Genome Biol..

[46] Wang J., Vasaikar S., Shi Z., Greer M., Zhang B. (2017). A More Comprehensive, Powerful, Flexible and Interactive Gene Set Enrichment Analysis Toolkit. Nucleic Acids Res..

[47] Clark M.J., Homer N., O’Connor B.D., Chen Z., Eskin A., Lee H., Merriman B., Nelson S.F. (2010). U87MG Decoded: The Genomic Sequence of a Cytogenetically Aberrant Human Cancer Cell Line. PLoS Genet..

[48] Wang X., Chen J., Liu Y., You C., Mao Q. (2013). Mutant TP53 Enhances the Resistance of Glioblastoma Cells to Temozolomide by Up-Regulating O6-Methylguanine DNA-Methyltransferase. Neurol. Sci..

[49] Fenstermaker R.A., Ciesielski M.J., Castiglia G.J. (1998). Tandem Duplication of the Epidermal Growth Factor Receptor Tyrosine Kinase and Calcium Internalization Domains in A-172 Glioma Cells. Oncogene.

[50] Fan X., Aalto Y., Sanko S.G., Knuutila S., Klatzmann D., Castresana J.S. (2002). Genetic Profile, PTEN Mutation and Therapeutic Role of PTEN in Glioblastomas. Int. J. Oncol..

[51] Kinashi Y., Ikawa T., Takahashi S. (2020). The Combined Effect of Neutron Irradiation and Temozolomide on Glioblastoma Cell Lines with Different MGMT and P53 Status. Appl. Radiat. Isot..

[52] Perazzoli G., Prados J., Ortiz R., Caba O., Cabeza L., Berdasco M., Gonzalez B., Melguizo C. (2015). Temozolomide Resistance in Glioblastoma Cell Lines: Implication of MGMT, MMR, P-Glycoprotein and CD133 Expression. PLoS ONE.

[53] Othman N.S., Mohd Azman D.K. (2022). Andrographolide Induces G2/M Cell Cycle Arrest and Apoptosis in Human Glioblastoma DBTRG-05MG Cell Line via ERK1/2/c-Myc/P53 Signaling Pathway. Molecules.

[54] Yang S.H., Wang S.M., Syu J.P., Chen Y., Wang S.D., Peng Y.S., Kuo M.F., Kung H.N. (2014). Andrographolide Induces Apoptosis of C6 Glioma Cells via the ERK-P53-Caspase 7-PARP Pathway. Biomed. Res. Int..

[55] Yang S.L., Kuo F.H., Chen P.N., Hsieh Y.H., Yu N.Y., Yang W.E., Hsieh M.J., Yang S.F. (2017). Andrographolide Suppresses the Migratory Ability of Human Glioblastoma Multiforme Cells by Targeting ERK1/2-Mediated Matrix Metalloproteinase-2 Expression. Oncotarget.

[56] Chang C.Y., Pan P.H., Li J.R., Ou Y.C., Wang J.D., Lao S.L., Chen W.Y., Wang W.Y., Chen J.C. (2020). Aspirin Induced Glioma Apoptosis through Noxa Upregulation. Int. J. Mol. Sci..

[57] Navone S.E., Guarnaccia L., Cordiglieri C., Crisa F.M., Caroli M., Locatelli M., Schisano L., Rampini P., Miozzo M., Verde N.L. (2018). Aspirin Affects Tumor Angiogenesis and Sensitizes Human Glioblastoma Endothelial Cells to Temozolomide, Bevacizumab, and Sunitinib, Impairing Vascular Endothelial Growth Factor-Related Signaling. World Neurosurg..

[58] Pozzoli G., Marei H.E., Althani A., Boninsegna A., Casalbore P., Lionel L.M., Lanzilli G., Zonfrillo M., Petrucci G., Rocca B. (2019). Aspirin Inhibits Cancer Stem Cells Properties and Growth of Glioblastoma Multiforme through Rb1 Pathway Modulation. J. Cell. Physiol..

[59] Pozzoli G., Petrucci G., Navarra P., Marei H.E., Cenciarelli C. (2019). Aspirin Inhibits Proliferation and Promotes Differentiation of Neuroblastoma Cells via P21(Waf1) Protein up-Regulation and Rb1 Pathway Modulation. J. Cell. Mol. Med..

[60] Zhao Y., Kang J.H., Yoo K.C., Kang S.G., Lee H.J., Lee S.J. (2021). K-RAS Acts as a Critical Regulator of CD44 to Promote the Invasiveness and Stemness of GBM in Response to Ionizing Radiation. Int. J. Mol. Sci..

[61] Peng X., Wu M., Liu W., Guo C., Zhan L., Zhan X. (2020). miR-502-5p Inhibits the Proliferation, Migration and Invasion of Gastric Cancer Cells by Targeting SP1. Oncol. Lett..

[62] Shi H.Z., Wang D.N., Ma L.N., Zhu H. (2020). MicroRNA-362 Inhibits Cell Growth and Metastasis in Glioblastoma by Targeting MAPK1. Eur. Rev. Med. Pharmacol. Sci..

[63] Zhan L., Yang J., Liu Y., Cheng Y., Liu H. (2021). MicroRNA miR-502-5p Inhibits Ovarian Cancer Genesis by Downregulation of GINS Complex Subunit 2. Bioengineered.

[64] Ahmed E.A., Rajendran P., Scherthan H. (2022). The microRNA-202 as a Diagnostic Biomarker and a Potential Tumor Suppressor. Int. J. Mol. Sci..

[65] Buechner J., Tomte E., Haug B.H., Henriksen J.R., Lokke C., Flaegstad T., Einvik C. (2011). Tumour-Suppressor microRNAs Let-7 and Mir-101 Target the Proto-Oncogene MYCN and Inhibit Cell Proliferation in MYCN-Amplified Neuroblastoma. Br. J. Cancer.

[66] Xia L., Tan S., Zhou Y., Lin J., Wang H., Oyang L., Tian Y., Liu L., Su M., Wang H. (2018). Role of the NFκB-Signaling Pathway in Cancer. Onco Targets Ther..

[67] Steven A., Seliger B. (2016). Control of CREB Expression in Tumors: From Molecular Mechanisms and Signal Transduction Pathways to Therapeutic Target. Oncotarget.

[68] Sapio L., Salzillo A., Ragone A., Illiano M., Spina A., Naviglio S. (2020). Targeting CREB in Cancer Therapy: A Key Candidate or One of Many? An Update. Cancers.

[69] Ghafouri-Fard S., Khoshbakht T., Hussen B.M., Baniahmad A., Taheri M., Samsami M. (2022). A Review on the Role of NR2F1-AS1 in the Development of Cancer. Pathol. Res. Pract..

[70] Grimaldi G., Rajendra S., Matthews J. (2018). The Aryl Hydrocarbon Receptor Regulates the Expression of TIPARP and Its Cis Long Non-Coding RNA, TIPARP-AS1. Biochem. Biophys. Res. Commun..

[71] Hu Y., Wang X., Li C., Jiao L., Du Y. (2021). LINC01783 Accelerated Tongue Squamous Cell Carcinoma Progression via Inhibiting miR-199b-5p. J. Cell. Mol. Med..

[72] Li J., Li D., Zhang X., Li C., Zhu F. (2021). Long Noncoding RNA SLC9A3-AS1 Increases E2F6 Expression by Sponging microRNA-486-5p and Thus Facilitates the Oncogenesis of Nasopharyngeal Carcinoma. Oncol. Rep..

[73] Wang Z., Ran R., Zhang S., Zhou W., Lv J., Ma C., Zhang H. (2023). The Role of Long Non-Coding RNA HCG18 in Cancer. Clin. Transl. Oncol..

[74] Liu K., Liu J., Bo Q.F. (2019). MFI2-AS1 Regulates the Aggressive Phenotypes in Glioma by Modulating MMP14 via a Positive Feedback Loop. Eur. Rev. Med. Pharmacol. Sci..

[75] Guo H., Wu L., Yang Q., Ye M., Zhu X. (2015). Functional Linc-POU3F3 Is Overexpressed and Contributes to Tumorigenesis in Glioma. Gene.

[76] Lang H.L., Hu G.W., Chen C., Liu Y., Tu W., Lu Y.M., Wu L., Xu G.H. (2017). Glioma Cells Promote Angiogenesis through the Release of Exosomes Containing Long Non-Coding RNA POU3F3. Eur. Rev. Med. Pharmacol. Sci..

[77] Lu X., Wang J., Wang W., Lu C., Qu T., He X., Liu X., Guo R., Zhang E. (2022). Copy Number Amplification and SP1-Activated lncRNA MELTF-AS1 Regulates Tumorigenesis by Driving Phase Separation of YBX1 to Activate ANXA8 in Non-Small Cell Lung Cancer. Oncogene.

[78] Chai J., Qin L., Zhang G., Hua P., Jin C. (2022). Long Non-Coding MELTF Antisense RNA 1 Promotes and Prognosis the Progression of Non-Small Cell Lung Cancer by Targeting miR-1299. Bioengineered.

[79] Ding L., Liu T., Qu Y., Kang Z., Guo L., Zhang H., Jiang J., Qu F., Ge W., Zhang S. (2021). lncRNA MELTF-AS1 Facilitates Osteosarcoma Metastasis by Modulating MMP14 Expression. Mol. Ther. Nucleic Acids.

[80] Lawrenson K., Song H., Tyrer J., Ramus S.J., Phelan C., Lee J., Wozniak E., Karevan R., Pharoah P.D., Ovarian Cancer Association Consortium (2012). Abstract 2928: Functional Effects of SNPs in Non-Coding RNAs at the 3q25 Ovarian Cancer Susceptibility Locus. Cancer Res..

[81] Gozgit J.M., Vasbinder M.M., Abo R.P., Kunii K., Kuplast-Barr K.G., Gui B., Lu A.Z., Molina J.R., Minissale E., Swinger K.K. (2021). PARP7 Negatively Regulates the Type I Interferon Response in Cancer Cells and Its Inhibition Triggers Antitumor Immunity. Cancer Cell..

[82] Xu J., Yu T., Yue Z., Lu X., Zhang Y., Wang L., Ahrling S.S., Smith M.R., Li Y.C., Matthews J. (2025). PARP7 Inhibition Stabilizes STAT1/STAT2 and Relieves Experimental Autoimmune Encephalomyelitis in Mice. Cell Rep..

[83] Zhang L., Cao J., Dong L., Lin H. (2020). TiPARP Forms Nuclear Condensates to Degrade HIF-1alpha and Suppress Tumorigenesis. Proc. Natl. Acad. Sci. USA.

[84] Wang J., Dong S., Zhang J., Jing D., Wang W., Dong L., Zhao Y. (2020). LncRNA NR2F1-AS1 Regulates miR-371a-3p/TOB1 Axis to Suppress Proliferation of Colorectal Cancer Cells. Cancer Biother. Radiopharm..

[85] Sosa M.S., Parikh F., Maia A.G., Estrada Y., Bosch A., Bragado P., Ekpin E., George A., Zheng Y., Lam H.M. (2015). NR2F1 Controls Tumour Cell Dormancy via SOX9- and RARbeta-Driven Quiescence Programmes. Nat. Commun..

[86] Huang X., Huang M., Chen M., Chen X. (2022). lncRNA SLC9A3-AS1 Promotes Oncogenesis of NSCLC via Sponging microRNA-760 and May Serve as a Prognosis Predictor of NSCLC Patients. Cancer Manag. Res..

[87] Xu X., Qing H., Jiang C., Zhao X., Wei J. (2025). Influence of the lncRNA SLC9A3-AS1 on Colon Cancer and the Biological Activities of Colon Cancer Cells. Discov. Onc..

[88] Lopez-Bertoni H., Kotchetkov I.S., Mihelson N., Lal B., Rui Y., Ames H., Fagundo-Lugo M., Cazares-Guerrero H., Quinones-Hinojosa A., Green J.J. (2020). A Sox2:miR-486-5p Axis Regulates Survival of GBM Cells by Inhibiting Tumor Suppressor Networks. Cancer Res..

[89] Sanchez Y., Segura V., Marin-Bejar O., Athie A., Marchese F.P., Gonzalez J., Bujanda L., Guo S., Matheu A., Huarte J. (2014). Genome-Wide Analysis of the Human P53 Transcriptional Network Unveils a lncRNA Tumour Suppressor Signature. Nat. Commun..

[90] Zhang A., Xu M., Mo Y.Y. (2014). Role of the lncRNA-P53 Regulatory Network in Cancer. J. Mol. Cell Biol..

[91] Wishart D.S., Knox C., Guo A.C., Shrivastava S., Hassanali M., Stothard P., Chang Z., Woolsey J. (2006). DrugBank: A Comprehensive Resource for in Silico Drug Discovery and Exploration. Nucleic Acids Res..

[92] Xia Y.F., Ye B.Q., Li Y.D., Wang J.G., He X.J., Lin X., Yao X., Ma D., Slungaard A., Hebbel R.P. (2004). Andrographolide Attenuates Inflammation by Inhibition of NF-kappaB Activation through Covalent Modification of Reduced Cysteine 62 of P50. J. Immunol..

[93] Nguyen V.S., Loh X.Y., Wijaya H., Wang J., Lin Q., Lam Y., Wong W.S., Mok Y.K. (2015). Specificity and Inhibitory Mechanism of Andrographolide and Its Analogues as Antiasthma Agents on NF-kappaB P50. J. Nat. Prod..

[94] Cherry E.M., Lee D.W., Jung J.U., Sitcheran R. (2015). Tumor Necrosis Factor-like Weak Inducer of Apoptosis (TWEAK) Promotes Glioma Cell Invasion through Induction of NF-**κ**B-Inducing Kinase (NIK) and Noncanonical NF-**κ**B Signaling. Mol. Cancer.

[95] Pflug K.M., Lee D.W., McFadden K., Herrera L., Sitcheran R. (2023). Transcriptional Induction of NF-**κ**B-Inducing Kinase by E2f4/5 Facilitates Collective Invasion of GBM Cells. Sci. Rep..

[96] Krauer F., Riesen M., Reveiz L., Oladapo O.T., Martinez-Vega R., Porgo T.V., Haefliger A., Broutet N.J., Low N. (2017). Zika Virus Infection as a Cause of Congenital Brain Abnormalities and Guillain-Barre Syndrome. Systematic Review. PLoS Med..

[97] Zhou C., Cheng M.L., He M.J., Liu Y., Li Y.Y., Xie D.Y., Chen L.S., Li D.Y., Deng Y.Q., Xu Y.P. (2026). MicroRNA-124-Targeted Recombinant Zika Virus: A Dual-Functional and Safe Candidate for Vaccination and Oncolytic Virotherapy. J. Virol..

[98] Retallack H., Di Lullo E., Arias C., Knopp K.A., Laurie M.T., Sandoval-Espinosa C., Leon W.R., Krencik R., Ullian E.M., Spatazza J. (2016). Zika Virus Cell Tropism in the Developing Human Brain and Inhibition by Azithromycin. Proc. Natl. Acad. Sci. USA.

[99] Chen J., Yang Y.F., Yang Y., Zou P., Chen J., He Y., Shui S.L., Cui Y.R., Bai R., Liang Y.J. (2018). AXL Promotes Zika Virus Infection in Astrocytes by Antagonizing Type I Interferon Signalling. Nat. Microbiol..

[100] Ojha C.R., Rodriguez M., Karuppan M.K., Laiperre J., Kashanchi F., El-Hage N. (2019). Toll-Like Receptor 3 Regulates Zika Virus Infection and Associated Host Inflammatory Response in Primary Human Astrocytes. PLoS ONE.

[101] Hutterer M., Knyazev P., Abate A., Reschke M., Maier H., Stefanova N., Knyazeva T., Barbieri V., Reindl M., Muigg A. (2008). Axl and Growth Arrest-Specific Gene 6 Are Frequently Overexpressed in Human Gliomas and Predict Poor Prognosis in Patients with Glioblastoma Multiforme. Clin. Cancer Res..

[102] Heiland D.H., Ravi V.M., Behringer S.P., Frenking J.H., Wurm J., Joseph K., Garrelfs N.W., Strahle J., Heynckes S., Grauvogel J. (2019). Tumor-Associated Reactive Astrocytes Aid the Evolution of Immunosuppressive Environment in Glioblastoma. Nat. Commun..

