# Supplementary material for: Transcriptomic Meta-Analysis and Functional Validation Identify Long Non-Coding RNAs as Modulators of Zika Virus-Mediated Oncolysis in Glioblastoma Multiforme Cell Lines
Source: Cells. 2026 Jun 15;15(12):1088. doi: 10.3390/cells15121088 (PMC13297335; doi:10.3390/cells15121088)
Supplement: Supplementary file 1 [file cells-15-01088-s001.zip › cells-4315692-supplementary.pdf]

SUPPLEMENTAL FIGURES:

1st Author	Manuscript Title	SRA ID	Number of Datasets	Zika Strain(s)	Sample Type	Sequencing Platform
Bonenfant	Asian Zika Virus Isolate Significantly Changes the Transcriptional Profile and Alternative RNA Splicing Events in a Neuroblastoma Cell Line	PRJNA630088	2	PRVABC59 MR766	SH-SY5Y Neuroblastoma cells	Illumina NextSeq 500
Zhu	Zika virus has oncolytic activity against glioblastoma stem cells	PRJNA399336	1	Dakar 41519	Glioblastoma stem cells (GSCs) and differentiated glioma cells (DGCs)	Illumina HiSeq 3000
Bulstrode	Myeloid cell interferon secretion restricts Zika flavivirus infection of developing and malignant human neural progenitor cells	PRJNA739733	1	PE243	E22 and E34 Glioblastoma cells	Illumina Novaseq 6000

Table S1. Overview of meta-analysis studies.

	Sequence 5' to 3'	Size of product
**HPRT Forward**	TGACACTGGCAAAACAATGCA	94
**HPRT Reverse**	GGTCCTTTTCACCAGCAAGCT	
**TIPARP_AS1 Forward**	TGCGACCCCAACTAAATCCT	136
**TIPARP_AS1 Reverse**	AGACTGCCCTTTGTACCGAA	

**NR2F1_AS1 Forward**	CCCCAGAGCTGCATCCTTAT	111
**NR2F1_AS1 Reverse**	TCCATAGTTGACATAGGCTTCC	
**MELTF_AS1 Forward**	TGGAAGAGGCGTTCAGAAGC	185
**MELTF_AS1 Reverse**	TTCTGAGCAGGGTGGAGAGG	
**SLC9A3_AS1 Forward**	CTGTTGGATGGAGGCCGTTA	157
**SLC9A3_AS1 Reverse**	CTTGTGCCCCAGAGTTTCCT	

Table S2. lncRNA primers used for quantitative real-time PCR.

A

Entrez ID	Symbol	Description	Predicted Expression
7412	VCAM1	vascular cell adhesion molecule 1	Upregulated
3383	ICAM1	intercellular adhesion molecule 1	Upregulated
1649	DDIT3	DNA damage inducible transcript 3	Upregulated
8638	OASL	2'-5'-oligoadenylate synthetase like	Upregulated
5971	RELB	RELB proto-oncogene, NF-kB subunit	Upregulated
2669	GABRA6	gamma-aminobutyric acid type A receptor subunit alpha6	Upregulated
54498	SMOX	spermine oxidase	Upregulated
90637	ZFAND2A	zinc finger AN1-type containing 2A	Upregulated
8767	RIPK2	receptor interacting serine/threonine kinase 2	Upregulated
51009	DERL2	derlin 2	Upregulated
54934	KANSL2	KAT8 regulatory NSL complex subunit 2	Upregulated
64782	AEN	apoptosis enhancing nuclease	Upregulated
9197	SLC33A1	solute carrier family 33 member 1	Upregulated
330	BIRC3	baculoviral IAP repeat containing 3	Upregulated
4189	DNAJB9	DnaJ heat shock protein family (Hsp40) member B9	Upregulated
3726	JUNB	JunB proto-oncogene, AP-1 transcription factor subunit	Upregulated
64089	SNX16	sorting nexin 16	Upregulated
50484	RRM2B	ribonucleotide reductase regulatory TP53 inducible subunit M2B	Upregulated
467	ATF3	activating transcription factor 3	Upregulated
27230	SERP1	stress associated endoplasmic reticulum protein 1	Upregulated
3669	ISG20	interferon stimulated exonuclease gene 20	Upregulated
51191	HERC5	HECT and RLD domain containing E3 ubiquitin protein ligase 5	Upregulated
221895	JAZF1	JAZF zinc finger 1	Upregulated
285636	RIMOC1	RAB7A interacting MON1-CCZ1 complex subunit 1	Upregulated
9709	HERPUD1	homocysteine inducible ER protein with ubiquitin like domain 1	Upregulated
4783	NFIL3	nuclear factor, interleukin 3 regulated	Upregulated
10140	TOB1	transducer of ERBB2, 1	Upregulated
4791	NFKB2	nuclear factor kappa B subunit 2	Upregulated
26511	CHIC2	cysteine rich hydrophobic domain 2	Upregulated
55254	TMEM29A	transmembrane protein 39A	Upregulated
4793	NFKBIB	NFKB inhibitor beta	Upregulated
6236	RRAD	RRAD, Ras related glycolysis inhibitor and calcium channel regulator	Upregulated
8878	SQSTM1	sequestosome 1	Upregulated
7494	XBP1	X-box binding protein 1	Upregulated
84919	PPP1R15B	protein phosphatase 1 regulatory subunit 15B	Upregulated
23471	TRAM1	translocation associated membrane protein 1	Upregulated
23764	MAFF	MAF bZIP transcription factor F	Upregulated
3656	IRAK2	interleukin 1 receptor associated kinase 2	Upregulated
7185	TRAF1	TNF receptor associated factor 1	Upregulated
2908	NR3C1	nuclear receptor subfamily 3 group C member 1	Upregulated
81788	NUAK2	NUAK family kinase 2	Upregulated
7538	ZFP36	ZFP36 ring finger protein	Upregulated
3659	IRF1	interferon regulatory factor 1	Upregulated
150274	HSCB	HscB mitochondrial iron-sulfur cluster cochaperone	Upregulated
9592	IER2	immediate early response 2	Upregulated
4790	NFKB1	nuclear factor kappa B subunit 1	Upregulated
56927	GPR108	G protein-coupled receptor 108	Upregulated
220213	OTUD1	OTU deubiquitinase 1	Upregulated
4792	NFKBIA	NFKB inhibitor alpha	Upregulated
23092	ARHGAP26	Rho GTPase activating protein 26	Upregulated
27113	BBC3	BCL2 binding component 3	Upregulated
65084	TMEM135	transmembrane protein 135	Upregulated
373	TRIM23	tripartite motif containing 23	Upregulated
6352	CCL5	C-C motif chemokine ligand 5	Upregulated
51307	FAM53C	family with sequence similarity 53 member C	Upregulated
2181	ACSL3	acyl-CoA synthetase long chain family member 3	Upregulated
51278	IER5	immediate early response 5	Upregulated
51061	TXNDC11	thioredoxin domain containing 11	Upregulated
64651	CSRNP1	cysteine and serine rich nuclear protein 1	Upregulated
83667	SESN2	sestrin 2	Upregulated
3433	IFIT2	interferon induced protein with tetratricopeptide repeats 2	Upregulated
6811	STX5	syntaxin 5	Upregulated
23645	PPP1R15A	protein phosphatase 1 regulatory subunit 15A	Upregulated
4084	MXD1	MAX dimerization protein 1	Upregulated
9619	ABCG1	ATP binding cassette subfamily G member 1	Upregulated
1263	PLK3	polo like kinase 3	Upregulated
9572	NR1D1	nuclear receptor subfamily 1 group D member 1	Upregulated
694	BTG1	BTG anti-proliferation factor 1	Upregulated
51030	TVP23B	trans-golgi network vesicle protein 23 homolog B	Upregulated
9095	TBX19	T-box transcription factor 19	Upregulated
10802	SEC24A	SEC24 homolog A, COPII coat complex component	Upregulated
1326	MAP3K8	mitogen-activated protein kinase kinase kinase 8	Upregulated
8780	RIOK3	RIO kinase 3	Upregulated
55602	CDKN2AIP	CDKN2A interacting protein	Upregulated
27289	RND1	Rho family GTPase 1	Upregulated
7358	UGDH	UDP-glucose 6-dehydrogenase	Upregulated
9546	APBA3	amyloid beta precursor protein binding family A member 3	Upregulated
96459	FNIP1	folliculin interacting protein 1	Upregulated
64764	CREB3L2	cAMP responsive element binding protein 3 like 2	Upregulated
4616	GADD45B	growth arrest and DNA damage inducible beta	Upregulated
3717	JAK2	Janus kinase 2	Upregulated
3638	INSIG1	insulin induced gene 1	Upregulated
1294	COL7A1	collagen type VII alpha 1 chain	Upregulated
6347	CCL2	C-C motif chemokine ligand 2	Upregulated
6319	SCD	stearoyl-CoA desaturase	Upregulated
1052	CEBPD	CCAAT enhancer binding protein delta	Upregulated
2643	GCH1	GTP cyclohydrolase 1	Upregulated
43	ACHE	acetylcholinesterase (Yt blood group)	Upregulated
51726	DNAJB11	DnaJ heat shock protein family (Hsp40) member B11	Upregulated
3309	HSPA5	heat shock protein family A (Hsp70) member 5	Upregulated
7873	MANF	mesencephalic astrocyte derived neurotrophic factor	Upregulated
5228	PGF	placental growth factor	Upregulated
8553	BHLHE40	basic helix-loop-helix family member e40	Upregulated
9601	PDIA4	protein disulfide isomerase family A member 4	Upregulated
10525	HYOU1	hypoxia up-regulated 1	Upregulated
79174	CRELD2	cysteine rich with EGF like domains 2	Upregulated
23753	SDF2L1	stromal cell derived factor 2 like 1	Upregulated
9719	ADAMTSL2	ADAMTS like 2	Upregulated
55715	DOK4	docking protein 4	Downregulated
10220	GDF11	growth differentiation factor 11	Downregulated
4001	LMNB1	lamin B1	Downregulated
400506	KNOP1	lysine rich nucleolar protein 1	Downregulated
85449	KIAA1755	KIAA1755	Downregulated
51280	GOLM1	golgi membrane protein 1	Downregulated
2583	B4GALNT1	beta-1,4-N-acetyl-galactosaminyltransferase 1	Downregulated
56135	PCDHAC1	protocadherin alpha subfamily C, 1	Downregulated
1463	NCAN	neurocan	Downregulated
2137	EXTL3	exostosin like glycosyltransferase 3	Downregulated
56098	PCDHGC4	protocadherin gamma subfamily C, 4	Downregulated
23240	TMEM131L	transmembrane 131 like	Downregulated
83879	CDCA7	cell division cycle associated 7	Downregulated
5764	PTN	pleiotrophin	Downregulated
152002	XXYLT1	xyloside xylosyltransferase 1	Downregulated
6941	TCF19	transcription factor 19	Downregulated
7112	TMPO	thymopoietin	Downregulated
899	CCNF	cyclin F	Downregulated
57462	MYORG	myogenesis regulating glycosidase	Downregulated
79888	LPCAT1	lysophosphatidylcholine acyltransferase 1	Downregulated
9498	SLC4A8	solute carrier family 4 member 8	Downregulated
150946	GAREM2	GRB2 associated regulator of MAPK1 subtype 2	Downregulated
2969	GTF2I	general transcription factor Iii	Downregulated
25758	KIAA1549L	KIAA1549 like	Downregulated
9917	FAM20B	FAM20B glycosaminoglycan xylosylkinase	Downregulated
51435	SCARA3	scavenger receptor class A member 3	Downregulated
65998	ZFTA	zinc finger translocation associated	Downregulated
57556	SEMA6A	semaphorin 6A	Downregulated
79966	SCD5	stearoyl-CoA desaturase 5	Downregulated
50512	PODXL2	podocalyxin like 2	Downregulated
10236	HNRNPR	heterogeneous nuclear ribonucleoprotein R	Downregulated
2899	GRIK3	glutamate ionotropic receptor kainate type subunit 3	Downregulated
92370	PXYLP1	2-phosphoxylose phosphatase 1	Downregulated
54058	C21orf58	chromosome 21 open reading frame 58	Downregulated
9315	NREP	neuronal regeneration related protein	Downregulated
8914	TIMELESS	timeless circadian regulator	Downregulated
5098	PCDHGC3	protocadherin gamma subfamily C, 3	Downregulated
5738	PTGFRN	prostaglandin F2 receptor inhibitor	Downregulated
3488	IGFBP5	insulin like growth factor binding protein 5	Downregulated
84913	ATOH8	atonal bHLH transcription factor 8	Downregulated
9806	SPOCK2	SPARC (osteonectin), cwcv and kazal like domains proteoglycan 2	Downregulated
7168	TPM1	tropomyosin 1	Downregulated
1844	DUSP2	dual specificity phosphatase 2	Downregulated
338382	RAB7B	RAB7B, member RAS oncogene family	Downregulated
27253	PCDH17	protocadherin 17	Downregulated
115207	KCTD12	potassium channel tetramerization domain containing 12	Downregulated

B

Entrez ID	Gene Type	Symbol	Description	Predicted Expression in GBM
124909347	ncRNA	LOC124909347	Uncharacterized	Upregulated
124907970	ncRNA	LOC124907970	Uncharacterized	Upregulated
105372436	ncRNA	LOC105372436	Uncharacterized	Upregulated
441094	ncRNA	NR2F1-AS1	NR2F1 antisense RNA 1	Upregulated
105370361	ncRNA	LINC03032	Long intergenic non-protein coding RNA 3032	Upregulated
100287227	ncRNA	TIPARP-AS1	TIPARP antisense RNA 1	Upregulated
100287216	ncRNA	SH3RF3-AS1	SH3RF3 antisense RNA 1	Upregulated
107983990	ncRNA	LOC107983990	Uncharacterized	Upregulated
105370449	ncRNA	LOC105370449	Uncharacterized	Upregulated
100996712	miscRNA	SRGAP2D	Pseudogene	Upregulated
644961	miscRNA	ACTG1P20	Pseudogene	Upregulated
100132057	miscRNA	PDE4DIPP6	Pseudogene	Upregulated
124904006	ncRNA	LOC124904006	Uncharacterized	Upregulated
105375914	ncRNA	LOC105375914	Uncharacterized	Upregulated
100132147	ncRNA	LINC01783	long intergenic non-protein coding RNA 1783	Upregulated
284600	ncRNA	LOC284600	Uncharacterized	Downregulated
155400	miscRNA	NSUN5P1	Pseudogene	Downregulated
105375304	ncRNA	SNX10-AS1	SNX10 antisense RNA 1	Downregulated
100129482	miscRNA	ZNF37BP	Pseudogene	Downregulated
130872	miscRNA	AHSA2P	Pseudogene	Downregulated
100132249	ncRNA	LOC100132249	Uncharacterized	Downregulated
55073	miscRNA	LRRC37A4P	Pseudogene	Downregulated
100288152	ncRNA	SLC9A3-AS1	SLC9A3 antisense RNA 1	Downregulated
442075	ncRNA	EMC3-AS1	EMC3 antisense RNA 1	Downregulated
414777	ncRNA	HCG18	HLA complex group 18	Downregulated
100507057	ncRNA	MELTF-AS1	MELTF antisense RNA 1	Downregulated
100506421	ncRNA	PANTR1	POU3F3 adjacent non-coding transcript 1	Downregulated
29774	miscRNA	POM121L9P	Pseudogene	Downregulated
503638	miscRNA	DUXAP9	Pseudogene	Downregulated
401303	miscRNA	ZNF815P	Pseudogene	Downregulated
100532732	ncRNA	MSH5-SAPCD1	MSH5-SAPCD1 readthrough (NMD candidate)	Downregulated

Table S3. Differentially Expressed Genes. Lists of the coding genes (A) significantly dysregulated in at least three out of the four datasets and non-coding genes (B) consistently dysregulated between the two glioblastoma studies. Genes highlighted in blue (A) denote the seven genes dysregulated in all four datasets. Genes highlighted in yellow (B) denote those selected for functional literature review.

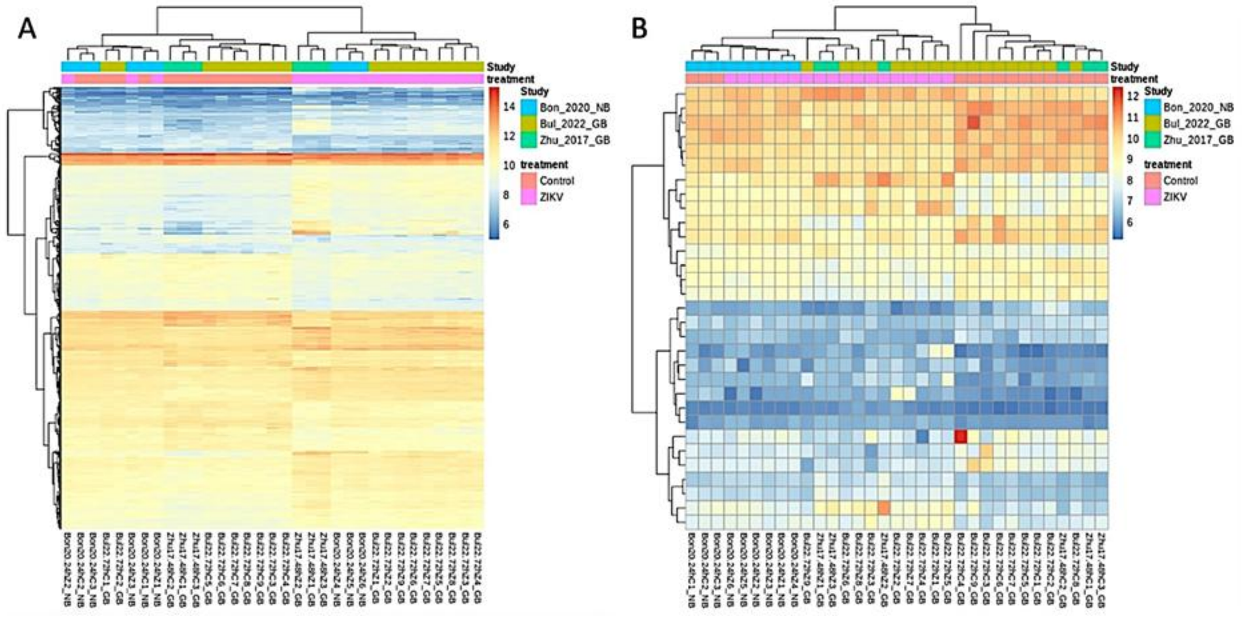

Figure S1. Heatmaps of coding and non-coding genes. Heatmaps depicting the transcriptional patterns of differentially expressed coding genes (A) and non-coding genes (B) between ZIKV-infected and control glioblastoma and neuroblastoma across the three studies.

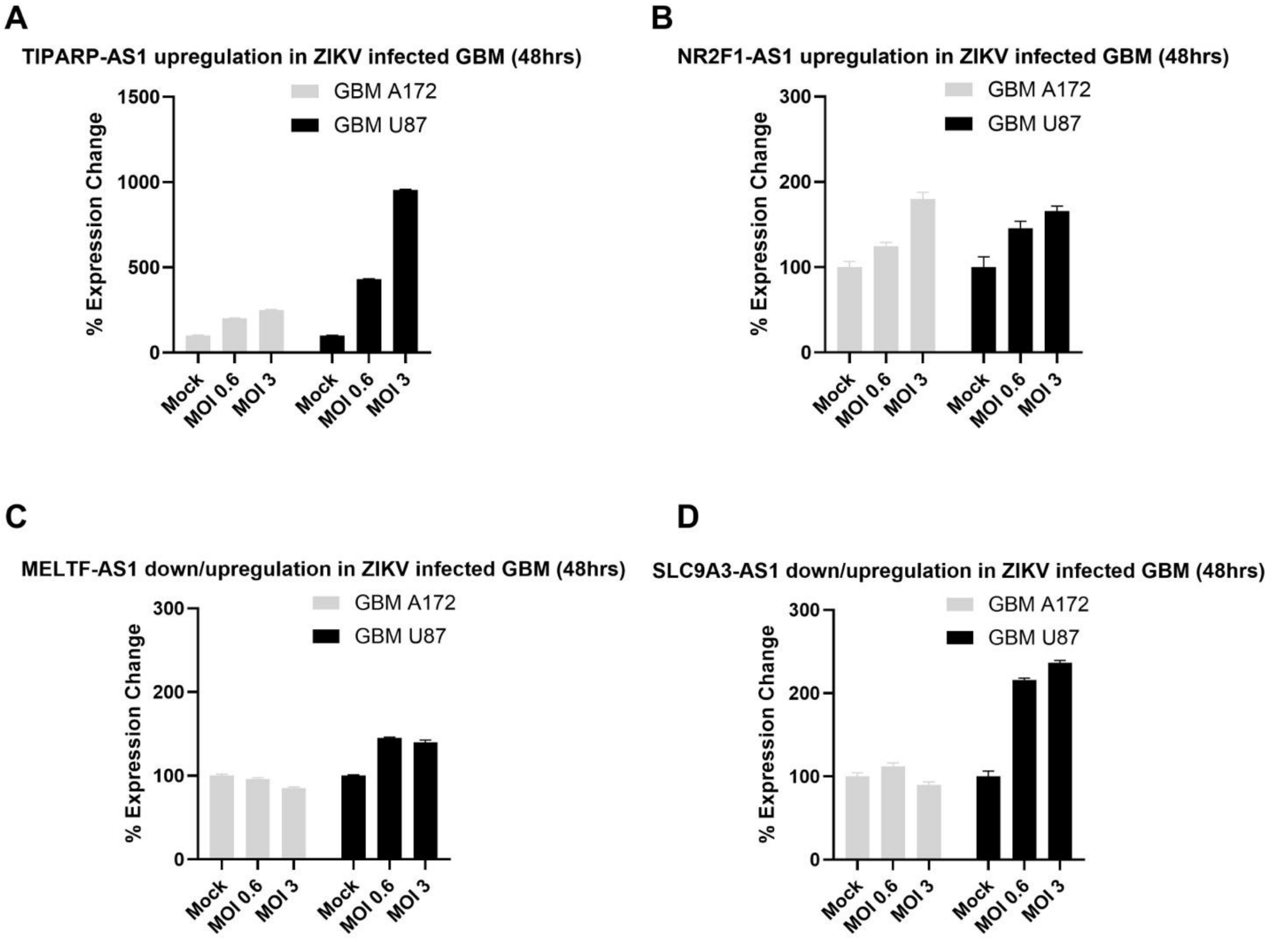

Figure S2. Differential Expression of Candidate lncRNAs in GBM A172 and U87 Cell Lines Following ZIKV infection. Both cell lines were subjected to mock treatment or infected with ZIKV at an MOI of 0.6 or 3. Expression levels of all four candidate lncRNAs were evaluated at 48 hours post-infection by quantitative real-time PCR (qPCR), with *HPRT* as the housekeeping reference gene. **(A)** Expression of *TIPARP-AS1* in GBM A172 and U87. **(B)** Expression of *NR2F1-AS1* under the same conditions. **(C)** Expression of *MELTF-AS1* under the same conditions. **(D)** Expression of *SLC9A3-AS1* under the same conditions. Expression is represented as a percentage of the mock control, with data representing the mean ± SD of qPCR technical duplicates. Statistical significance was assessed by one-way matching ANOVA for all gene expression evaluations; no comparison reached statistical significance.

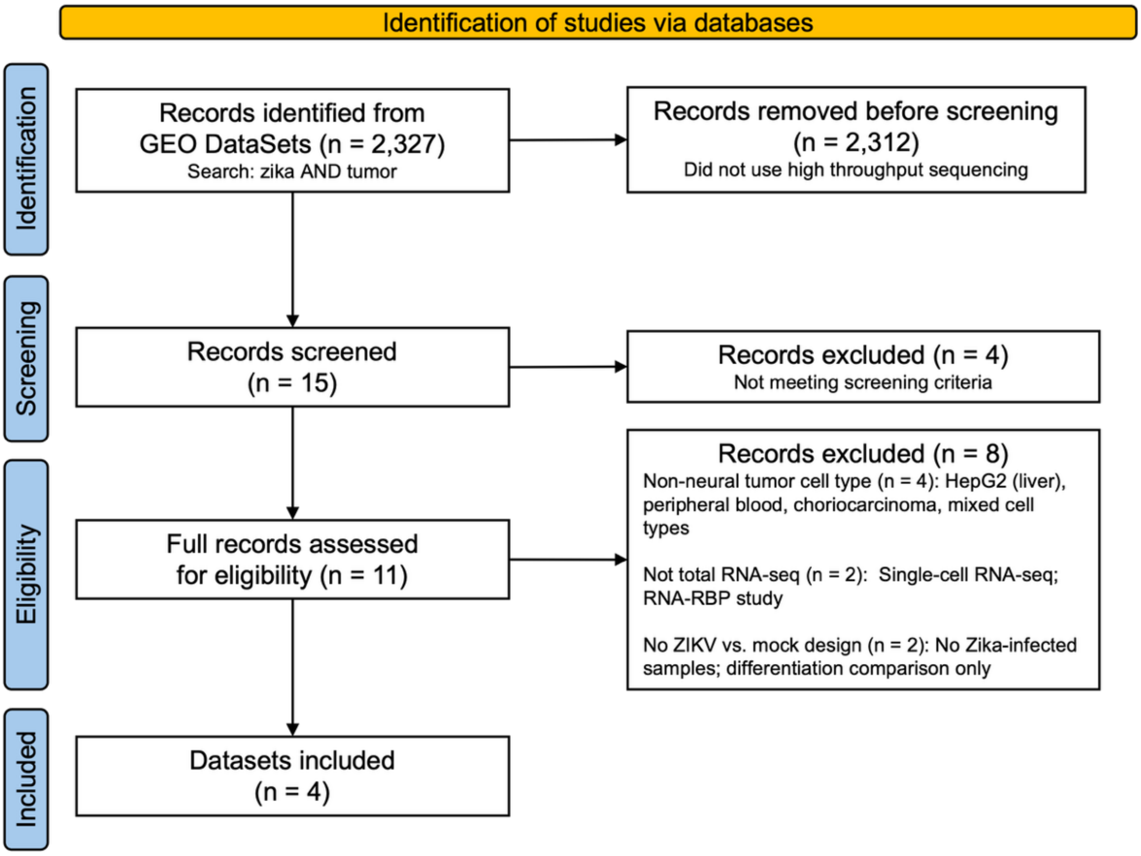

Figure S3. PRISMA flowchart for GEO DataSets search and transcriptomic dataset selection. Flowchart depicting the identification, screening, and eligibility assessment of publicly available RNA-seq datasets retrieved from the Gene Expression Omnibus (GEO) DataSets database (search terms: *zika* AND *tumor*). Abbreviations: RNA-RBP, RNA–RNA-binding protein interaction study.
